# The N terminus of α-synuclein dictates fibril formation

**DOI:** 10.1073/pnas.2023487118

**Published:** 2021-08-27

**Authors:** Ryan P. McGlinchey, Xiaodan Ni, Jared A. Shadish, Jiansen Jiang, Jennifer C. Lee

**Affiliations:** ^a^Laboratory of Protein Conformation and Dynamics, Biochemistry and Biophysics Center, National Heart, Lung, and Blood Institute, Bethesda, MD 20892;; ^b^Laboratory of Membrane Proteins and Structural Biology, Biochemistry and Biophysics Center, National Heart, Lung, and Blood Institute, Bethesda, MD 20892

**Keywords:** α-synuclein, N-terminal truncation, amyloid, Parkinson’s disease, cryo-EM

## Abstract

The generation of α-synuclein (α-syn) truncations from incomplete proteolysis plays a significant role in the pathogenesis of Parkinson’s disease. It is well established that C-terminal truncations exhibit accelerated aggregation and serve as potent seeds in fibril propagation. In contrast, mechanistic understanding of N-terminal truncations remains ill defined. Previously, we found that disease-related C-terminal truncations resulted in increased fibrillar twist, accompanied by modest conformational changes in a more compact core, suggesting that the N-terminal region could be dictating fibril structure. Here, we examined three N-terminal truncations, in which deletions of 13-, 35-, and 40-residues in the N terminus modulated both aggregation kinetics and fibril morphologies. Cross-seeding experiments showed that out of the three variants, only ΔN13-α-syn (14‒140) fibrils were capable of accelerating full-length fibril formation, albeit slower than self-seeding. Interestingly, the reversed cross-seeding reactions with full-length seeds efficiently promoted all but ΔN40-α-syn (41–140). This behavior can be explained by the unique fibril structure that is adopted by 41–140 with two asymmetric protofilaments, which was determined by cryogenic electron microscopy. One protofilament resembles the previously characterized bent β-arch kernel, comprised of residues E46‒K96, whereas in the other protofilament, fewer residues (E61‒D98) are found, adopting an extended β-hairpin conformation that does not resemble other reported structures. An interfilament interface exists between residues K60‒F94 and Q62‒I88 with an intermolecular salt bridge between K80 and E83. Together, these results demonstrate a vital role for the N-terminal residues in α-syn fibril formation and structure, offering insights into the interplay of α-syn and its truncations.

## Significance

α-Synuclein truncations are prevalent in Parkinson’s disease. While C-terminal truncations are known to accelerate α-synuclein aggregation, the understanding of how N-terminal truncations influence fibril formation lags behind. Here, we show that the removal of N-terminal residues outside the canonical amyloid core weakens the fibril seeding efficiency and hinders propagation. Remarkably, a unique, asymmetric amyloid core is revealed by cryogenic electron microscopy in the N-terminally truncated 41–140 variant, highlighting the strong influence of the N terminus in dictating fibril structure. This work offers insights into the interplay of truncations and full-length proteins; thus, changes in their relative levels could prove to be beneficial for molecular intervention.

Amyloid formation of α-synuclein (α-syn) is a pathological feature of Parkinson’s disease (PD), multiple-system atrophy (MSA), and dementia with Lewy bodies (1, 2). An abundant presynaptic protein (3), α-syn is 140 amino acids in length with a putative biological function in aiding the exocytosis of synaptic vesicles (4–6), in which the first 89 N-terminal residues fold into a helical structure upon membrane association (7). In its disease-associated, aggregated amyloid state, residues 37 through 97 adopt β-sheet structure (8), which overlaps with the lipid-binding domain. Notably, both N- and C-terminal α-syn truncations are associated with PD (9). So far, N-terminally truncated (ΔN) α-syn variants 5‒140, 39‒140, 65‒140, 66‒140, 68‒140, and 71‒140 and C-terminally truncated (ΔC) α-syn variants, 1‒101, 1‒103, 1‒115, 1‒122, 1‒124, 1‒135, and 1‒139 have been found in brains of PD patients (10–12).

α-Syn truncations originate from incomplete degradation, which has been attributed to various cytosolic (13–15) and lysosomal proteases (16, 17). In fact, ∼60% of the abovementioned truncations can be assigned to cleavages by lysosomal asparagine endopeptidase (AEP), cathepsin (Cts) D, CtsB, and CtsL (15–17). Removal of the C terminus (residues 104–140) is shown to accelerate fibril formation both in vitro and in vivo (18–25). On the other hand, perplexing behaviors of ΔN-variants have been documented; while deleting the first 20 residues has minimal perturbation, the removal of either the first 10 or 30 residues slows aggregation kinetics (26). Nevertheless, the influence of N-terminal residues on α-syn aggregation has been shown by both insertion [tandem repeat of residues 9–30 (27)] and deletion [Δ36–42 (28) and Δ52–55 (29)] mutants, in which fibril formation can be completely impeded.

**Fig. 1.**
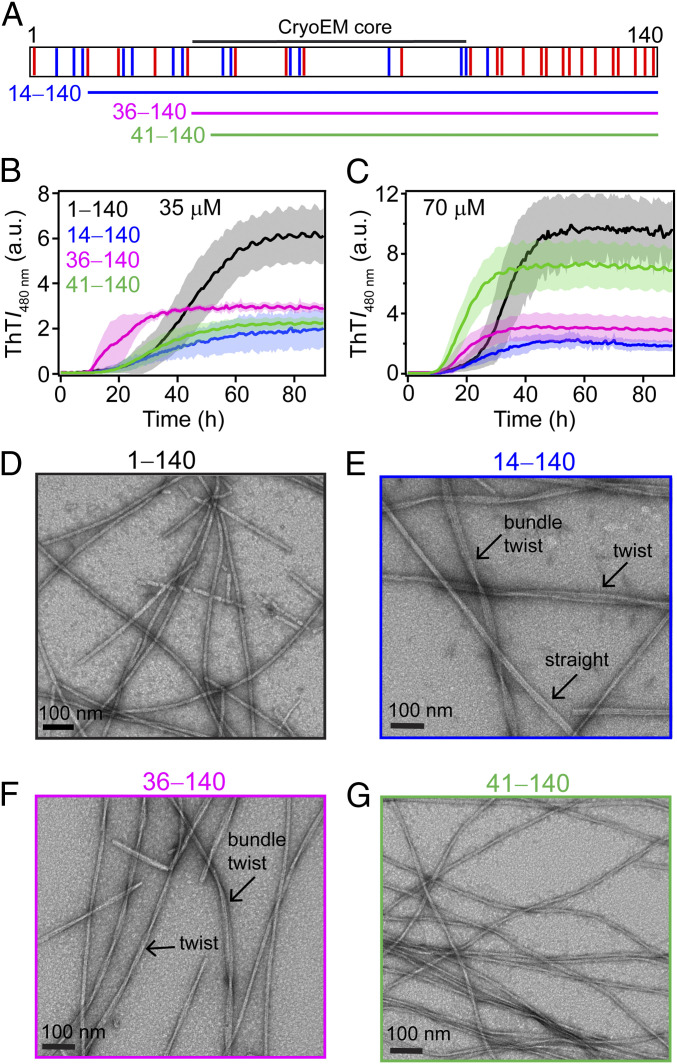
Aggregation of ΔN-α-syns. (*A*) Schematic representation of α-syn primary sequence (residues 1–140), showing basic (blue) and acidic (red) residues. Underlined regions correspond to truncations used in this study: 14‒140 (blue), 36‒140 (magenta), and 41‒140 (green). (*B* and *C*) Comparison of aggregation kinetics monitored by ThT fluorescence at 37 °C. [α-Syn] = 35 µM (*B*) and 70 µM (*C*) with [ThT] = 10 µM in 20 mM NaPi, 140 mM NaCl, pH 7.4. The solid line and shaded region represent the mean and SD, respectively (*n* ≥ 4). Representative TEM images of (*D*) 1‒140, (*E*) 14‒140, (*F*) 36‒140, and (*G*) 41‒140 were taken at 35 µM. Different fibril polymorphs observed are noted. Additional fields of view are shown in *SI Appendix*, Figs. S3–S5.

Recently, structure determination by cryogenic electron microscopy (cryo-EM) has revealed fibril structures for full-length α-syn (1–140) (24, 30–32), C-terminal truncations (24, 33), phosphorylated Y39 (34), and PD-related mutants, E46K (35, 36), H50Q (37), and A53T (38). One striking feature of these fibrils is the eclectic mix of structures, often termed as fibril polymorphism. In fact, it was recently shown that different conformational strains of α-syn fibrils are present in PD and MSA patients (39, 40). The outstanding question still remains as to how the same polypeptide chain can produce such a vast number of polymorphic structures. While there are significant structural differences, some features of α-syn fibrils are conserved. All fibrils are formed from a twisting pair of protofilaments with the exception of a H50Q polymorph, which is composed of a single filament. A kernel motif of a bent β-arch appears in all structures. Also, at least one inter- or intramolecular salt bridge between a Lys and Glu is revealed in each structure (24, 30–38, 40), which is not surprising given that there are numerous possibilities for salt bridges between the 14 Lys, 8 Glu, and 2 Asp residues located throughout the first 100 residues in the sequence (Fig. 1*A* and *SI Appendix*, Fig. S1). Generally, residues between 37 and 97 constitute the fibril core with a few exceptions that involve additional residues in the N terminus, which include phosphorylated Y39 fibrils with an extended core of 1–100 (34) and two polymorphs of 1–140 showing interactions of N-terminal β-strands (residues 14–24) (30). Fibrils derived from brains of MSA patients also indicate additional involvement of the N-terminal region extending to residue 14 (40). Due to the contribution of N-terminal residues in these structures and the fact that C-terminal truncations resulted in modest conformational changes, we hypothesize that N-terminal residues play a greater role in influencing fibril structure.

Here, we sought to understand the role of the N terminus in α-syn fibril formation by removing different N-terminal residues and evaluating their effects on aggregation kinetics, fibril structure, and propagation. Three ∆N-terminal constructs (14‒140, 36‒140, and 41‒140) have been examined, in which the first 13-, 35-, and 40-residues in the N terminus were deleted (Fig. 1*A*). We specifically chose these sites based on the locations of native Gly residues, which allows us to generate native sequences (i.e., no overhang) upon Tobacco Etch Virus (TEV) protease cleavage of the hexahistidine affinity tag, which facilitates facile protein purification. All three ∆N-α-syn exhibited different aggregation kinetics and distinct fibril ultrastructural features as determined by thioflavin-T (ThT) fluorescence and transmission electron microscopy (TEM), respectively. In cross-seeding experiments, both fibrillar 36‒140 and 41‒140 did not seed the full-length (1‒140) protein, while 14‒140 fared better but less efficient than self-seeding, supportive of the significant impact of removing N-terminal residues in fibril structure. The reverse reaction involving full-length seeds showed that fibril formation of 14‒140 and 36‒140 but not 41‒140 could be accelerated. This observation is explained by the fibril structure adopted by 41–140, which was determined by cryo-EM to an overall resolution of 3.2 Å. Unlike any currently known α-syn structure, the amyloid core is formed by two asymmetric protomers with different amino acid chain lengths, adopting an extended β-hairpin (E61‒D98) and the bent β-arch kernel (E46‒K96) with a large nonpolar interfilament interface (442 Å^2^) stabilized by an intermolecular salt bridge between K80 and E83. Collectively, these results establish the important role of N-terminal residues in fibril formation and structure.

## Results

### N-Terminal Truncations Impact Fibril Formation Kinetics.

To assess the role that the N terminus plays in fibril assembly, three ∆N-α-syn proteins were generated: 14–140, 36–140, and 41–140 (Fig. 1*A*). Their aggregation propensities were first compared to the full-length protein at two protein concentrations (35 and 70 µM) using ThT, an extrinsic fluorophore that increases in intensity upon binding to amyloid structures (41). At 35 µM (Fig. 1*B*), 1‒140 aggregation proceeded with a typical sigmoidal curve with a time to reach half maximum intensity (*t*_1/2_) of 44 ± 4 h, which was reduced to 33 ± 4 h at 70 µM (Fig. 1*C*). While greater variations in kinetics were observed at 35 µM, they were largely consistent with trends seen at 70 µM, with all ΔN-α-syn showing overall faster aggregation kinetics compared to 1‒140 with 36‒140 being the fastest, followed by 41‒140 and 14‒140 (*SI Appendix*, Fig. S2). Specifically, t_1/2_ values were 25 ± 3, 20 ± 2, and 19 ± 2 h for 70 µM 14‒140, 36‒140, and 41‒140, respectively.

Interestingly, lower final ThT intensities were consistently observed for the ΔN-α-syn variants. To determine whether the observed ThT intensity differences are due to relative amounts of aggregated proteins, sodium dodecyl sulfate–polyacrylamide gel electrophoresis (SDS-PAGE) analysis was performed after ultracentrifugation (*SI Appendix*, Fig. S2). Densitometric analysis of the aggregation reactions revealed that only for 14‒140 does the lower final ThT signal correlate with a reduced amount of aggregated proteins (50% compared to 85% full-length). In contrast, at least 90% of both 36‒140 and 41‒140 are in the pelleted fraction, which is comparable to 1‒140, suggestive of ThT sensitivity to fibril polymorphism.

### N-Terminal Truncations Impact Fibril Morphologies.

Negative-stain TEM images taken postaggregation show fibril differences at the ultrastructural level (Fig. 1 *D–G*). As previously reported (24), rod-shaped fibrils ∼10 nm in width were observed for the full-length protein (Fig. 1*D*). In the cases of 14‒140 and 36‒140, many different fibril morphologies were seen, including rod-like, twisted, and laterally associated fibrils (Fig. 1 *E* and *F* and *SI Appendix*, Figs. S3 and S4). The twisted fibrils have varying helical pitches ranging from ∼54 to 300 nm. For 41‒140, the main species are twisted fibrils with a helical pitch ∼53 nm with a minor population of fibril bundles (Fig. 1*G* and *SI Appendix*, Fig. S5). These twisted fibrils are not seen for 1‒140.

### N-Terminal Truncations Impact Fibril Resistance to Limited Proteolysis.

To evaluate the protease-resistant structured cores of the ΔN-α-syn fibrils, limited proteolysis was performed using a broad-spectrum protease, proteinase K (PK). SDS-PAGE analysis revealed greater resistance for 14‒140 and 36‒140 compared to degradation profiles of 1‒140 (*SI Appendix*, Fig. S6). In contrast, 41‒140 was more protease sensitive, especially at the higher PK concentrations. Liquid chromatography–mass spectrometry analysis (*SI Appendix*, Table S2) indicated that there is a consistent C-terminal cutting site at residues Y125/E126 (in which / denotes cut site) for all constructs. For 36‒140 and 41‒140, another cut site at L113/E114 is also revealed. In the N terminus, 1‒140 revealed cleavages at A18/A19, A19/E20, and A30/G31 with the smallest PK-resistant core being residues 31‒125. For 14‒140, 36‒140, and 41‒140, their smallest cores are 36‒125, 36‒113, and 41‒113, respectively. An additional cleavage site at H50/G51 was also observed for 41‒140, resulting in a 51‒125 fragment. It is interesting to note that cut sites E35/G36, H50/G51, and L113/E114 are not observed in the full-length protein, suggesting that the structures of full-length and ΔN-α-syn fibrils are distinct.

### Evaluation of N-Terminal Truncations on Cellular Viability.

The cytotoxicity of 1‒140, 14‒140, 36‒140, and 41‒140 α-syn fibrils were examined in two different cell lines: human SH-SY5Y neuroblastoma and rat dopaminergic N27 cells. Cell viability was assessed by mitochondrial activity after 48 h treatment with fibrils (1 μM). Fibrils were prepared by ultracentrifugation, and the pelleted fibrils were quantified prior to treatment. Using a standard 3-(4,5-dimethylthiazol-2-yl)-2,5-diphenyltetrazolium bromide assay, impaired mitochondrial activity was consistently observed from all fibril treatments compared to the buffer control (*SI Appendix*, Fig. S7); however, the uncertainty in our measurements precluded the evaluation whether there were significant differences among the ΔN-variants and the full-length fibrils for either cell line. Based on these results, these ΔN-fibrils have comparable cytotoxicity to cells under the conditions examined.

### N-Terminal Residues Are Essential in Fibril Propagation.

**Fig. 2.**
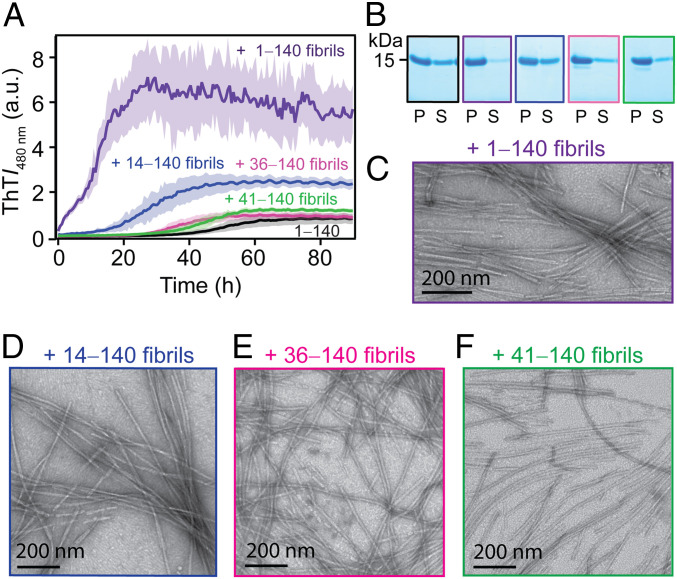
Cross-seeding kinetics of 1‒140 with ΔN-α-syn fibrils. (*A*) Aggregation kinetics of 1‒140 in the absence (black) and presence of preformed 1‒140 (purple), 14‒140 (blue), 36‒140 (magenta), or 41‒140 (green) fibrils (5% mol/mol) monitored by ThT fluorescence ([1‒140] = 35 µM and [ThT] = 3.5 µM in 20 mM NaPi, 140 mM NaCl, pH 7.4 at 37 °C). The solid line and shaded region represent the mean and SD, respectively (*n* ≥ 4). Additional seeding data (10% mol/mol) are shown in *SI Appendix*, Fig. S8. (*B*) SDS-PAGE analysis of pelleted (P) and soluble (S) protein by ultracentrifugation postaggregation. (*C‒F*) Representative TEM images of 1‒140 fibrils formed in the presence of preformed ΔN-α-syn fibrils. Colored as in *A*.

We next asked whether these ΔN-α-syn fibrils could cross-propagate or seed soluble full-length protein (Fig. 2*A* and *SI Appendix*, Fig. S8). As a reference, self-seeding by preformed 1‒140 fibrils (5%) accelerated the aggregation of the full-length protein by abrogating the lag phase and enhancing the final ThT signal. This result is recapitulated at a higher seed concentration (10%, *SI Appendix*, Fig. S8). By comparison, only the addition of 14‒140 fibrils stimulated 1‒140 aggregation by shortening the lag time and increasing the final ThT signal, whereas in the presence of either 36‒140 or 41‒140 fibrils, 1‒140 exhibited indistinguishable kinetics to that of itself alone. SDS-PAGE analysis indicated less pelleted material after ultracentrifugation for 14‒140 seeded reactions, supporting that 14‒140 fibrils are inferior seeds for 1‒140 (Fig. 2*B*). While 14‒140 appears to cross-seed on the basis of aggregation kinetics analysis, the resulting TEM images of the 1‒140 fibrils formed under all conditions are highly similar to each other (Fig. 2 *C*–*F*), providing no evidence for propagation of the 14‒140 fibril morphologies.

**Fig. 3.**
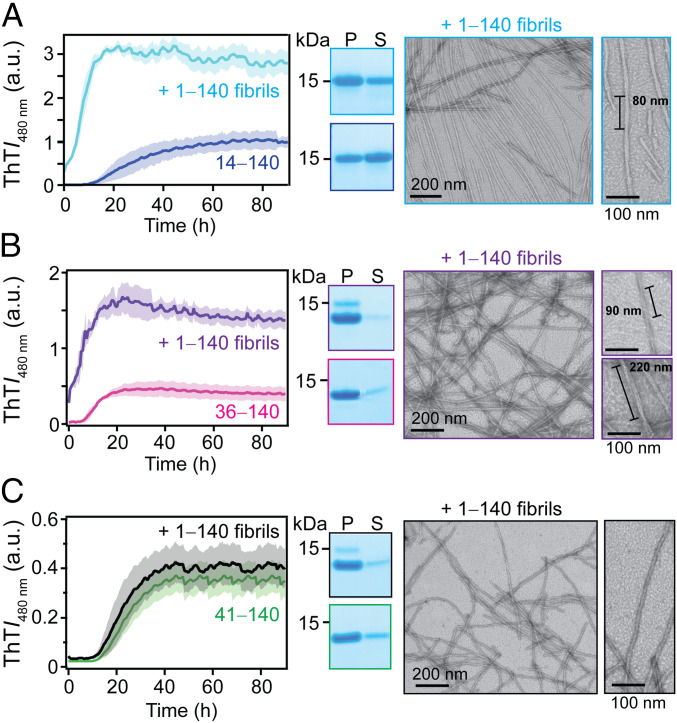
Cross-seeding kinetics of ΔN-α-syn with 1‒140 fibrils. Aggregation kinetics of 14‒140 (*A*), 36‒140 (*B*), and 41‒140 (*C*) in the absence and presence of 1‒140 fibrils (5% mol/mol) monitored by ThT fluorescence (*Left*). ([ΔN-α-syn] = 35 µM and [ThT] = 3.5 µM in 20 mM NaPi, 140 mM NaCl, pH 7.4 at 37 °C). The solid line and shaded region represent the mean and SD, respectively (*n* ≥ 4). Additional seeding data (10% mol/mol) for 41‒140 are shown in *SI Appendix*, Fig. S9. SDS-PAGE analysis of pelleted (P) and soluble (S) proteins by ultracentrifugation postaggregation (*Middle*). Representative TEM images of ΔN-α-syn fibrils formed in the presence of preformed 1‒140 fibrils (*Right*). Expanded views show different twisted fibril polymorphs.

Having established that 36‒140 and 41‒140 fibrils cannot cross-seed the full-length soluble protein, we investigated whether the reverse reaction would also be prohibitive, which would further substantiate the importance of N-terminal residues in fibril propagation. Efficient seeding was observed in the presence of full-length fibrils for both 14‒140 and 36‒140, with lag phases abolished and higher final ThT signals (Fig. 3 *A* and *B*). In contrast, 1‒140 fibrils had no measurable effect on the behavior of 41‒140 (Fig. 3*C*), indicating that seeding did not occur. Even upon doubling the seed concentration, the full-length fibril is unable to seed 41‒140, suggestive of structural incompatibility (*SI Appendix*, Fig. S9). Postaggregation SDS-PAGE analysis revealed that while 14‒140 has a higher level of aggregated materials compared to the unseeded control, a similar amount of pelleted protein was obtained for 36‒140, cautioning that higher ThT signals do not necessarily correlate to fibril amounts.

This cross-seeding by full-length fibrils of 14‒140 was corroborated by TEM images taken postaggregation, which revealed more homogeneous fibrils with a half pitch of ∼80 nm (Fig. 3 *A*, *Right*), which are not characterized from those of the unseeded reaction (*SI Appendix*, Fig. S3). Interestingly, while the fibril morphology of 36‒140 remained heterogenous (Fig. 3 *B*, *Right*), the observed helical pitches of the twisted fibrils were ∼90 and 220 nm, which again are different from those of the unseeded reaction (*SI Appendix*, Fig. S4), indicating an effect of the full-length seeds on the growth of 36‒140 fibrils. It is evident that 1‒140 was not able to promote 41‒140 fibril growth since the fibrils are highly reminiscent (Fig. 3 *C*, *Right*) to those formed from the 41‒140 control (*SI Appendix*, Fig. S5).

Collectively, these cross-seeding experiments suggest that different mechanisms are at play depending on the fibril seeds and soluble proteins used. Since 14‒140, but neither 36‒140 or 41‒140 fibrils, could enhance 1‒140 aggregation, residues between 14 and 35 could be important in the productive recruitment of full-length protein. Alternatively, the removal of the N-terminal 35 residues could completely change the fibril structure that is favored only by ΔN-variants. However, when using seeds with an intact N terminus, only 41‒140 failed to template, which would indicate that residues 36 through 40 are more important in either scenario, in the replication of the full-length structure and the formation of a different fibril structure.

### Fibril Structure of 41‒140 Reveals Asymmetric Protofilaments.

**Fig. 4.**
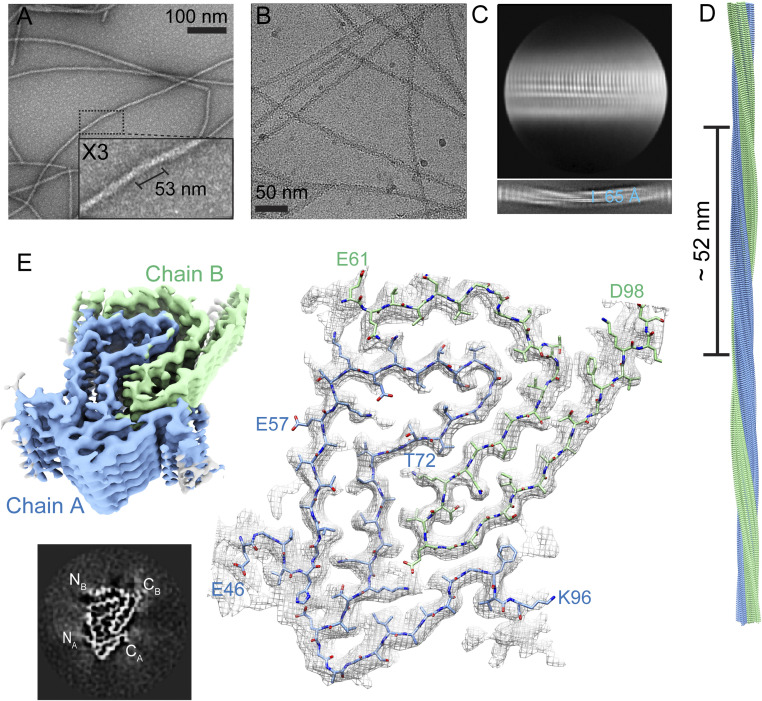
Cryo-EM structure of 41‒140 fibrils. (*A*) Representative negative-stain TEM image of 41‒140 fibrils. (*B*) Representative cryo-EM micrograph of 41‒140 fibrils. (*C*) (*Top*) Representative two-dimensional (2D) class average showing a near-parallel conformation between two protofilaments. (*Bottom*) 2D class average in a larger box size. The fibril diameter is as indicated. (*D*) Fibril side view depicted with a half pitch of 52 nm. (*E*, *Top Left*) Cryo-EM density map with two protofilaments, chains A and B, colored blue and green, respectively. (*Bottom Left*) Cross-section along the *x*-*y* plane of the density map of the three-dimensional reconstruction. The resolvable N- and C-terminal regions are labeled. (*Right*) Model of 41‒140. The cross-sectional view shows the fit of the model in the density map. The fibril contains two asymmetric protomers, chains A (blue) and B (green), consisting of residues E46–K96 and E61–D98, respectively.

To gain an understanding to our cross-seeding observations, we sought to obtain fibril structural information by cryo-EM. Out of the three ∆N-constructs studied, 41‒140 fibrils had the most homogenous population, and thus, the fibril structure of 41‒140 was successfully determined by cryo-EM at near-atomic resolution (*SI Appendix*, Table S3). The twisted fibrils seen in negative-stain TEM (Fig. 4*A*) are recapitulated in the cryo-EM micrograph (Fig. 4*B*). Two-dimensional (2D) class averages indicate a fibril width of ∼6.5 nm (Fig. 4*C*). Interestingly, the density map of cryo-EM three-dimensional (3D) reconstruction shows two asymmetrical protofilaments intertwining into a left-handed helix with the helical twist of −1.64° with a half pitch ∼52 nm (Fig. 4*D*), similar to our negative-stain TEM (Fig. 4*A*). The two protofilaments, designated as chains A and B (Fig. 4*E*, green and blue), stack along the axis with a helical rise of 4.8 Å as determined by the layer lines from the power spectrum of the micrographs (*SI Appendix*, Fig. S10). A cross-sectional density map of the fibril is also shown in Fig. 4 *E*, *Bottom Left*.

The fibril structure was determined at an overall resolution of 3.2 Å (Fig. 4 *E*, *Right* and *SI Appendix*, Fig. S11), with clear side-chain density that permitted unambiguous de novo atomic model building. A different number of residues was resolved for the two asymmetric protofilaments, comprising residues E46‒K96 and E61‒D98 for chains A and B, respectively. Chain A folds into a β-strand–rich architecture including the β-arch kernel (E57–T72) that is present in other full-length α-syn structures (31–33). Chain B adopts a dramatically different β-hairpin conformation. Unlike chain A, the lack of density for N-terminal residues G41–K60 suggests conformational flexibility in this region for this protomer. We note that during image processing, a minor second conformation of 41‒140 was also observed, showing additional density at the N terminus of chain B (*SI Appendix*, Fig. S12). While specific residue assignment was not possible, it likely represents residues between G41 and K60. This extra density also results in compaction of residues E46–H50 in chain A that is otherwise more extended in the main polymorph. Similar to published structures (31–33), there is no visible density for the C terminus (residues 99–140), highlighting its disordered nature in the fibrillar state.

### Key Interactions in 41‒140 Fibrils.

**Fig. 5.**
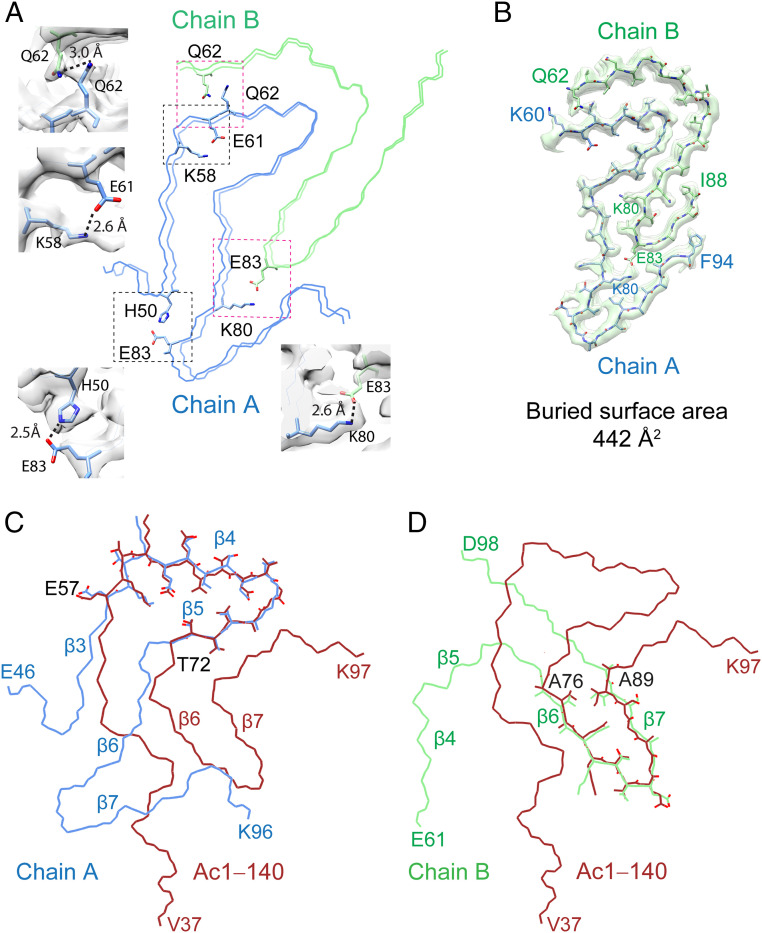
Key interactions of 41‒140 and comparison to Ac1‒140 fibrils. (*A*) Polypeptide backbone of 41‒140 structure showing key side-chain interactions. Black boxes highlight intramolecular interactions of a salt bridge between K58 and E61 and hydrogen-bonding between H50 and E83 in chain A (blue). Magenta boxes indicate intermolecular interactions of a salt bridge between K80 (chain A, blue) and E83 (chain B, green) and hydrogen-bonding between Q62 and Q62 from each protomer. (*B*) The interface between chain A and B is composed of K60‒F94 and Q62‒I88, respectively. The buried surface area is calculated to be 442 Å^2^. Overlay of Ac1‒140 (PDB 6OSJ, red) with (*C*) chain A (blue) and (*D*) chain B (green) with β-strand assignments based on the Ac1‒140 structure. Residues E57–T72 (chain A) adopt the conserved β-arch kernel with identical side-chain orientations as the full-length protein. For chain B, a minimal conserved region is found between residues A76 and A89 with their side chains shown.

Key interactions that define the structure are highlighted in Fig. 5*A*. The β-arch motif in chain A is stabilized by a previously documented intramolecular salt bridge between K58 and E61 (24, 31, 37, 38) and hydrogen-bonding between H50 and E83. There is a large interface between the two protofilaments, consisting of residues K60‒F94 (chain A) and Q62‒I88 (chain B). An intermolecular salt bridge is formed by K80 in chain A and E83 in chain B, an interaction not observed in other structures (*SI Appendix*, Fig. S1). Additional hydrogen-bonding is found between the two Q62 residues. This interface (Fig. 5*B*) has a calculated buried surface area of 442 Å^2^ with an estimated total binding energy of −7.87 kcal/mol which is nearly twice of that of the steric zipper between residues H50 and E57 in the full-length protein (239 Å^2^ and −4.49 kcal/mol). We note that K80 from chain B is found buried at the interface, which was also found in the hydrophobic core of the E46K fibril structure, in which it is interacting with a resolved water molecule (36). Although a water molecule is not resolved at the current resolution, there is sufficient space to fit one near the ε-amino group. Thus, we surmise that the ε-amino group in 41‒140 is also hydrogen-bonding with a water molecule, similar to that of the E46K fibril structure.

### Comparison of 41‒140 to Acetylated Full-Length α-syn.

A comparison of chains A and B of 41‒140 to the acetylated (Ac) 1‒140 protomer (Protein Data Bank [PDB] code 6OSJ) is shown in Fig. 5 *C* and *D*, respectively. A highly consistent β-arch motif is found between residues E57 through T72 for chain A and Ac1‒140 (denoted as β4 and β5 strands in the full-length structure). Clear differences in the region spanning residues G73 to D98 are observed between the two protomers, in which chain A in 41‒140 moves away in order to accommodate the insertion of a portion of chain B, which brings E83 of chain B into close proximity to form an intermolecular salt bridge with K80 of chain A. This interaction is unique to 41‒140 and has not been observed in other structures (*SI Appendix*, Fig. S1) (24, 31–33). In contrast, K80 forms an intramolecular salt bridge with E46 in Ac1–140, bringing β6 and β2 close together (*SI Appendix*, Fig. S13). In the Ac1–140 structure, β7 is folded back and packs against β6.

The overlay of chain B with Ac1‒140 shows striking conformational differences. There are only 13 overlapping residues, A76 to A89, which span strands β6 and β7. This extended hairpin conformation composed of residues G68 to V95 appears unique to this structure thus far. Collectively, these results support the idea that N-terminal residues modulate the mechanism of amyloid assembly and contribute to the molecular interactions that define fibril structure.

## Discussion

Fibril polymorphism is a defining feature of pathological amyloids, yet mechanistic insight into how multiple structures are generated from a given polypeptide chain is still an open question. In this work, we explored the impact of the N terminus on α-syn fibril formation by using a series of N-terminal truncations. The removal of 13-, 35-, and 40-residues from the N terminus–influenced aggregation behaviors (Fig. 1), and all three truncations have distinguishing ultrastructural features different from those exhibited by the full-length α-syn fibrils (Fig. 1 *C–F* and *SI Appendix*, Figs. S3–S5). Structure determination of 41‒140 by cryo-EM revealed a fibril conformation in which two asymmetric protofilaments with different lengths of ordered residues interwind to form a twisted fibril (Fig. 4). A large interface is formed between residues K60 and F94 (chain A) and Q62 and I88 (chain B), and the protomers are oriented in an N-to-C/N-to-C geometry (Fig. 5). An intermolecular salt bridge forms between K80 and E83, exemplifying the ability of the polypeptide chain to utilize the many available Lys and Glu residues as stabilizing factors (Fig. 1*A*). Including this work, there are currently a total of five unique intramolecular (K45–E57, K58–E57, K58–E61, K80–E46, and K96–E61) and six intermolecular (K45–E46, K45–E57, K58–E35, K60–E28, K80–E61, and K80–E83) Lys-Glu salt bridges found in α-syn fibril structures (*SI Appendix*, Fig. S1).

Since the N-terminal region (residues 1 through 36) is unresolved and thus presumed to be largely disordered in most full-length α-syn fibril structures, the impact of its removal on the 41‒140 structure was initially surprising. However, recent work has demonstrated the participation of N-terminal residues in two 1‒140 polymorphs in which residues V16 through E20 form a hydrophobic steric zipper with residues S87 through A91 (30). Also, fibrils derived from MSA patients showed a larger core comprising residues 14–99, with an extended N terminus forming a cross-β hairpin (40). Even our own work on C-terminally truncated Ac1–103 α-syn suggested the presence of additional N-terminal residues (24). Taken together, the N terminus plays a pivotal role in fibril assembly.

Using cross-fibril propagation experiments, we conclusively show the importance of the N terminus in α-syn fibril formation. As the N terminus is removed, ΔN-α-syn fibrils were shown to be poor seeds for soluble full-length α-syn (Fig. 2). This is noteworthy because the removal of C-terminal residues has the opposing effect in which it creates potent seeds and can efficiently propagate soluble full-length α-syn (17, 24). That said, the addition of 14‒140 fibrils did result in a lag time reduction, suggesting that residues 15 through 40 play a more crucial role. However, due to the unique fibril structure that is adopted by 41‒140 (Fig. 4), we speculate that this fibril structure is inaccessible to 1‒140 even when a template is provided. This result is surprising given that the structurally resolved core only involves residues 46 and beyond. Moreover, it suggests that the presence of the first 40 residues is prohibitive for propagation. While it is unknown whether 36‒140 also adopts a different fibril structure, it is evident that it behaves more like 41‒140. Thus, we propose that 1‒140 and 14‒140 fibrils are more alike compared to 36‒140 and 41‒140, which may be more similar to each other.

From a mechanistic standpoint, two recent studies also have highlighted the involvement of N-terminal residues in fibril formation. Specifically, residues 1–11 (42) and 1–12 (43) are identified in monomer addition to the ends of seeds (i.e., elongation) and monomer binding to the fibril surface (i.e., secondary nucleation), respectively. These results are in agreement with our cross-propagation experiments using ΔN-α-syn fibrils, in which the effect of the first 13 residues is clearly documented (Fig. 2). However, our data contradicts the conclusion that intermolecular contacts from residues 1 through 35 are essential as lag phases of both 14–140 and 36–140 can be bypassed by the addition of 1–140 seeds (Fig. 3), which indicate that monomers lacking either 1–13 or 1–35 can add to full-length seeds and form fibrils. Furthermore, since 41‒140 could not be seeded by 1‒140 fibrils, it points to the involvement of the intervening hydrophobic residues, ^3^^7^VLYV^40^, in facilitating the formation of the full-length structure. This postulate is supported by recent work on a Δ36–42 α-syn mutant (28), which suggested that this region modulates fibril formation. Collectively, these data affirm the strong influence of the N terminus and its interactions (or the lack thereof) in driving full-length α-syn amyloid formation. Lastly, the role of the C terminus should not be overlooked due to favorable electrostatic interactions with the N-terminal region, which is supported by the differences in limited-PK digestion among the variants compared to the full-length protein. Thus, it is imperative for future investigations to evaluate the effect of removing both N- and C-terminal residues and to address the interplay between the two termini in how a given α-syn structure is faithfully propagated.

With ∆N-α-syn modulating aggregation kinetics as well as fibril structure and propagation, a central question is whether these species are beneficial or detrimental in a cellular environment. It is known that both N- and C-terminal truncations are prevalent in both PD and healthy individuals (44), and some of their generation can be attributed to lysosomal protease activity (16, 17). While 41‒140 has not been identified in patients, it is related to another known truncation, 39‒140, which can be generated by CtsD activity (16). These proteases have long been implicated in α-syn turnover and given the opposing fibril propagation behaviors of ∆N- and ∆C-α-syn, it is imperative that future studies consider contributions from both fibril variants. With the enhanced seeding capabilities of C-terminal truncations (24), one could envision the presence of ∆N-α-syn potentially attenuating this deleterious effect. Such a scenario could occur in the lysosomal environment where levels of protease activity dictate the truncation generated. However, an absence of the N terminus could also reduce α-syn membrane binding, thus shifting the α-syn equilibrium in the cellular milieu and lead to dysfunction. This potential tug of war presents an interesting interplay between different truncation variants, providing future targets in tempering α-syn aggregation.

## Materials and Methods

Details regarding reagents, molecular cloning, protein expression and purification, aggregation kinetics, TEM, liquid chromatography–mass spectrometry, cell culture and cell viability assay, cryo-EM sample preparation, and data collection and processing as well as model and refinement are provided in *SI Appendix*.

## Supplementary Material

Supplementary File

## Data Availability

All data are made available within the article or *SI Appendix*. The cryo-EM density map for 41–140 was deposited in the Electron Microscopy Data Bank with the accession number EMD-23270. The associated atomic model of 41–140 was deposited in the Research Collaboratory for Structural Bioinformatics Protein Data Bank with the entry code 7LC9.

## References

[1] M. G. Spillantini, R. A. Crowther, R. Jakes, M. Hasegawa, M. Goedert, α-Synuclein in filamentous inclusions of Lewy bodies from Parkinson’s disease and dementia with Lewy bodies. Proc. Natl. Acad. Sci. U.S.A. 95, 6469–6473 (1998). 9600990 10.1073/pnas.95.11.6469 PMC27806

[2] K. Wakabayashi ., Accumulation of α-synuclein/NACP is a cytopathological feature common to Lewy body disease and multiple system atrophy. Acta Neuropathol. 96, 445–452 (1998). 9829807 10.1007/s004010050918

[3] K. Uéda ., Molecular cloning of cDNA encoding an unrecognized component of amyloid in Alzheimer disease. Proc. Natl. Acad. Sci. U.S.A. 90, 11282–11286 (1993). 8248242 10.1073/pnas.90.23.11282 PMC47966

[4] J. Burré ., α-Synuclein promotes SNARE-complex assembly in vivo and in vitro. Science 329, 1663–1667 (2010). 20798282 10.1126/science.1195227 PMC3235365

[5] J. Burré, M. Sharma, T. C. Südhof, α-Synuclein assembles into higher-order multimers upon membrane binding to promote SNARE complex formation. Proc. Natl. Acad. Sci. U.S.A. 111, E4274–E4283 (2014). 25246573 10.1073/pnas.1416598111 PMC4210039

[6] U. Kaur, J. C. Lee, Unroofing site-specific α-synuclein-lipid interactions at the plasma membrane. Proc. Natl. Acad. Sci. U.S.A. 117, 18977–18983 (2020). 32719116 10.1073/pnas.2006291117 PMC7430991

[7] C. C. Jao, B. G. Hegde, J. Chen, I. S. Haworth, R. Langen, Structure of membrane-bound α-synuclein from site-directed spin labeling and computational refinement. Proc. Natl. Acad. Sci. U.S.A. 105, 19666–19671 (2008). 19066219 10.1073/pnas.0807826105 PMC2605001

[8] M. Vilar ., The fold of α-synuclein fibrils. Proc. Natl. Acad. Sci. U.S.A. 105, 8637–8642 (2008). 18550842 10.1073/pnas.0712179105 PMC2438424

[9] A. W. Schmid, B. Fauvet, M. Moniatte, H. A. Lashuel, α-Synuclein post-translational modifications as potential biomarkers for Parkinson disease and other synucleinopathies. Mol. Cell. Proteomics 12, 3543–3558 (2013). 23966418 10.1074/mcp.R113.032730 PMC3861707

[10] J. F. Kellie ., Quantitative measurement of intact α-synuclein proteoforms from post-mortem control and Parkinson’s disease brain tissue by intact protein mass spectrometry. Sci. Rep. 4, 5797 (2014). 25052239 10.1038/srep05797 PMC4107347

[11] A. Ohrfelt ., Identification of novel α-synuclein isoforms in human brain tissue by using an online nanoLC-ESI-FTICR-MS method. Neurochem. Res. 36, 2029–2042 (2011). 21674238 10.1007/s11064-011-0527-x PMC3183298

[12] J. P. Anderson ., Phosphorylation of Ser-129 is the dominant pathological modification of α-synuclein in familial and sporadic Lewy body disease. J. Biol. Chem. 281, 29739–29752 (2006). 16847063 10.1074/jbc.M600933200

[13] W. Wang ., Caspase-1 causes truncation and aggregation of the Parkinson’s disease-associated protein α-synuclein. Proc. Natl. Acad. Sci. U.S.A. 113, 9587–9592 (2016). 27482083 10.1073/pnas.1610099113 PMC5003239

[14] R. Shams, N. L. Banik, A. Haque, Calpain in the cleavage of α-synuclein and the pathogenesis of Parkinson’s disease. Prog. Mol. Biol. Transl. Sci. 167, 107–124 (2019). 31601400 10.1016/bs.pmbts.2019.06.007 PMC8434815

[15] Z. Zhang ., Asparagine endopeptidase cleaves α-synuclein and mediates pathologic activities in Parkinson’s disease. Nat. Struct. Mol. Biol. 24, 632–642 (2017). 28671665 10.1038/nsmb.3433 PMC5871868

[16] R. P. McGlinchey, J. C. Lee, Cysteine cathepsins are essential in lysosomal degradation of α-synuclein. Proc. Natl. Acad. Sci. U.S.A. 112, 9322–9327 (2015). 26170293 10.1073/pnas.1500937112 PMC4522768

[17] R. P. McGlinchey ., C-terminal α-synuclein truncations are linked to cysteine cathepsin activity in Parkinson’s disease. J. Biol. Chem. 294, 9973–9984 (2019). 31092553 10.1074/jbc.RA119.008930 PMC6597809

[18] I. M. van der Wateren, T. P. J. Knowles, A. K. Buell, C. M. Dobson, C. Galvagnion, C-terminal truncation of α-synuclein promotes amyloid fibril amplification at physiological pH. Chem. Sci. (Camb.) 9, 5506–5516 (2018). 10.1039/c8sc01109e PMC6048717 30061982

[19] A. Ulusoy, F. Febbraro, P. H. Jensen, D. Kirik, M. Romero-Ramos, Co-expression of C-terminal truncated α-synuclein enhances full-length α-synuclein-induced pathology. Eur. J. Neurosci. 32, 409–422 (2010). 20704592 10.1111/j.1460-9568.2010.07284.x

[20] C. W. Liu ., A precipitating role for truncated α-synuclein and the proteasome in α-synuclein aggregation: Implications for pathogenesis of Parkinson disease. J. Biol. Chem. 280, 22670–22678 (2005). 15840579 10.1074/jbc.M501508200

[21] W. Hoyer, D. Cherny, V. Subramaniam, T. M. Jovin, Impact of the acidic C-terminal region comprising amino acids 109-140 on α-synuclein aggregation in vitro. Biochemistry 43, 16233–16242 (2004). 15610017 10.1021/bi048453u

[22] I. V. J. Murray ., Role of α-synuclein carboxy-terminus on fibril formation in vitro. Biochemistry 42, 8530–8540 (2003). 12859200 10.1021/bi027363r

[23] R. A. Crowther, R. Jakes, M. G. Spillantini, M. Goedert, Synthetic filaments assembled from C-terminally truncated α-synuclein. FEBS Lett. 436, 309–312 (1998). 9801138 10.1016/s0014-5793(98)01146-6

[24] X. Ni, R. P. McGlinchey, J. Jiang, J. C. Lee, Structural insights into α-synuclein fibril polymorphism: Effects of Parkinson’s disease-related C-terminal truncations. J. Mol. Biol. 431, 3913–3919 (2019). 31295458 10.1016/j.jmb.2019.07.001 PMC6733637

[25] Z. A. Sorrentino ., Physiological C-terminal truncation of α-synuclein potentiates the prion-like formation of pathological inclusions. J. Biol. Chem. 293, 18914–18932 (2018). 30327435 10.1074/jbc.RA118.005603 PMC6295729

[26] M. Terada ., The effect of truncation on prion-like properties of α-synuclein. J. Biol. Chem. 293, 13910–13920 (2018). 30030380 10.1074/jbc.RA118.001862 PMC6130941

[27] J. C. Kessler, J. C. Rochet, P. T. Lansbury Jr, The N-terminal repeat domain of α-synuclein inhibits β-sheet and amyloid fibril formation. Biochemistry 42, 672–678 (2003). 12534279 10.1021/bi020429y

[28] C. P. A. Doherty ., A short motif in the N-terminal region of α-synuclein is critical for both aggregation and function. Nat. Struct. Mol. Biol. 27, 249–259 (2020). 32157247 10.1038/s41594-020-0384-x PMC7100612

[29] V. V. Shvadchak, V. Subramaniam, A four-amino acid linker between repeats in the α-synuclein sequence is important for fibril formation. Biochemistry 53, 279–281 (2014). 24397337 10.1021/bi401427t

[30] R. Guerrero-Ferreira ., Two new polymorphic structures of human full-length α-synuclein fibrils solved by cryo-electron microscopy. eLife 8, e48907 (2019). 31815671 10.7554/eLife.48907 PMC6957273

[31] Y. Li ., Amyloid fibril structure of α-synuclein determined by cryo-electron microscopy. Cell Res. 28, 897–903 (2018). 30065316 10.1038/s41422-018-0075-x PMC6123497

[32] B. Li ., Cryo-EM of full-length α-synuclein reveals fibril polymorphs with a common structural kernel. Nat. Commun. 9, 3609 (2018). 30190461 10.1038/s41467-018-05971-2 PMC6127345

[33] R. Guerrero-Ferreira ., Cryo-EM structure of α-synuclein fibrils. eLife 7, 18 (2018). 10.7554/eLife.36402 PMC6092118 29969391

[34] K. Zhao ., Parkinson’s disease-related phosphorylation at Tyr39 rearranges α-synuclein amyloid fibril structure revealed by cryo-EM. Proc. Natl. Acad. Sci. U.S.A. 117, 20305–20315 (2020). 32737160 10.1073/pnas.1922741117 PMC7443891

[35] K. Zhao ., Parkinson’s disease associated mutation E46K of α-synuclein triggers the formation of a distinct fibril structure. Nat. Commun. 11, 2643 (2020). 32457390 10.1038/s41467-020-16386-3 PMC7250837

[36] D. R. Boyer ., The α-synuclein hereditary mutation E46K unlocks a more stable, pathogenic fibril structure. Proc. Natl. Acad. Sci. U.S.A. 117, 3592–3602 (2020). 32015135 10.1073/pnas.1917914117 PMC7035510

[37] D. R. Boyer ., Structures of fibrils formed by α-synuclein hereditary disease mutant H50Q reveal new polymorphs. Nat. Struct. Mol. Biol. 26, 1044–1052 (2019). 31695184 10.1038/s41594-019-0322-y PMC6907165

[38] Y. Sun ., Cryo-EM structure of full-length α-synuclein amyloid fibril with Parkinson’s disease familial A53T mutation. Cell Res. 30, 360–362 (2020). 32203130 10.1038/s41422-020-0299-4 PMC7118165

[39] M. Shahnawaz ., Discriminating α-synuclein strains in Parkinson’s disease and multiple system atrophy. Nature 578, 273–277 (2020). 32025029 10.1038/s41586-020-1984-7 PMC7066875

[40] M. Schweighauser ., Structures of α-synuclein filaments from multiple system atrophy. Nature 585, 464–469 (2020). 32461689 10.1038/s41586-020-2317-6 PMC7116528

[41] H. LeVine III, Quantification of β-sheet amyloid fibril structures with thioflavin T. Methods Enzymol. 309, 274–284 (1999). 10507030 10.1016/s0076-6879(99)09020-5

[42] X. Yang, B. Wang, C. L. Hoop, J. K. Williams, J. Baum, NMR unveils an N-terminal interaction interface on acetylated-α-synuclein monomers for recruitment to fibrils. Proc. Natl. Acad. Sci. U.S.A. 118, e2017452118 (2021). 33903234 10.1073/pnas.2017452118 PMC8106326

[43] P. Kumari ., Structural insights into α-synuclein monomer-fibril interactions. Proc. Natl. Acad. Sci. U.S.A. 118, e2012171118 (2021). 33649211 10.1073/pnas.2012171118 PMC7958257

[44] Z. A. Sorrentino, B. I. Giasson, The emerging role of α-synuclein truncation in aggregation and disease. J. Biol. Chem. 295, 10224–10244 (2020). 32424039 10.1074/jbc.REV120.011743 PMC7383394

