# Supplementary material for: The N terminus of α-synuclein dictates fibril formation
Source: Proc Natl Acad Sci U S A. 2021 Aug 27;118(35):e2023487118. doi: 10.1073/pnas.2023487118 (PMC8536336; doi:10.1073/pnas.2023487118)
Supplement: Supplementary File [file pnas.2023487118.sapp.pdf]

SI Appendix for

The N-terminus of α-synuclein dictates fibril formation

RP McGlinchey, X Ni, JA Shadish, J Jiang, JC Lee

Materials Methods

Table S1. List of primers used in the study.

Table S2. Mass spectrometry analysis of PK digestion

Table S3. Cryo-EM data collection, structure determination, and model statistics

Figure S1. Schematic representation of salt bridges found in α-syn fibril structures

Figure S2. Normalized aggregation kinetics and SDS-PAGE analysis of pellet *vs*. soluble fractions for ΔN-α-syn after aggregation

Figure S3. Additional negative-stain TEM images for 14–140

Figure S4. Additional negative-stain TEM images for 36–140

Figure S5. Additional negative-stain TEM images for 41–140

Figure S6. SDS-PAGE analysis of PK digestion

Figure S7. MTT assays for ΔN-α-syn fibrils

Figure S8. Cross-seeding of 1–140 by ΔN-α-syn fibrils at 10% seed concentration

Figure S9. Cross-seeding of 41–140 by 1–140 fibrils at 10% seed concentration

Figure S10 and **S11**. Cryo-EM data related analysis-power spectrum, local resolution estimation, gold standard FSC curves

Figure S12. Structure of a second conformation of 41–140

Figure S13. Intramolecular salt bridge in 1–140 fibril structure

MATERIAL AND METHODS

Reagents. Chemicals were obtained from Sigma unless otherwise noted.

Protein expression and purification. Plasmids for N-terminally truncated α-syn variants (residues 14–140, 36–140, and 41–140) were constructed using the primers listed in **Table S1** that generated sequences with cleavable N-terminal His-tags. These PCR amplified fragments were cloned into a pET21a(+) vector. For full-length α-syn, a pRK172 plasmid was used (1), and the protein was expressed and purified as previously described (2). The full length protein and truncations contain a silent mutation (TAT) at residue 136 to avoid the spontaneous mutation of Tyr-to-Cys (3). Constructs were verified by DNA sequencing (Psomagen, USA). Proteins were expressed in *E.coli* BL21(DE3) (New England BioLabs). Shaken cultures (1-L Luria Broth) were grown at 37 °C to an OD_{600} ~0.6 and then induced with 1 mM IPTG for 4 h. Cells were collected by centrifugation (6000 rpm, Sorvall SLC-6000) for 20 min at 4 °C and then resuspended in denaturing buffer (6 M guanidine hydrochloride, 100 mM NaCl, 100 mM K_2HPO_4, pH 7.5, and 10 mM imidazole) and incubated overnight at 4 °C. The resulting lysate was then spun at 30,000 rpm (Beckman Ti45) for 45 min at 4 °C. The pellet was discarded, and the supernatant was applied to a HisPrep FF 16/10 column (GE Healthcare) using an Äkta Pure chromatography system (GE Healthcare). The column was first washed with 3-column volumes of buffer A (8 M urea, 100 mM NaCl, 100 mM Na_2HPO_4, pH 7.5 and 10 mM imidazole). The protein was eluted using gradient method composed of buffer A and buffer B (8 M urea, 100 mM NaCl, 100 mM Na_2HPO_4, pH 7.5 and 500 mM imidazole). First, 1-column volume of 10% buffer B, followed by a linear gradient to 55% buffer B, and finally a step gradient to 100% buffer B. The eluted protein was buffer exchanged into 20 mM NaPi, 140 mM NaCl, pH 7.4 buffer using a PD-10 column (GE Healthcare).

For removal of the His-tag from 14–140, 36–140 and 41–140, a TEV protease was used. The His-tagged TEV protease in a pRK793 plasmid (Addgene Technologies, USA) was expressed in BL21-CodonPlus (DE3)-RIPL (Agilent Technologies, USA). A 20-L BioFlo 4500 fermenter (New Brunswick Scientific, Edison, NJ) was used for large scale fermentation and the protein was purified using the protocol described above with one minor modification of the addition of 0.1% PEI to precipitate DNA prior to affinity purification. Precipitated DNA was removed by centrifugation at 30,000 rpm (Beckman Ti45). For TEV protease activity, 1 mM DTT was added. All His-tagged proteins were initially incubated with protease (50 µL TEV (1 mg/mL) in 15 mL total volume) at 4 °C with cleavage efficiency monitored by SDS-PAGE and LC-MS analysis (NHLBI Biochemistry Core). Additional protease was added until complete removal of His-tags. After full cleavage, the protein sample was reapplied to nickel-nitrilotriacetic acid resin (Qiagen, NiNTA superflow agarose resin) and stirred at 4 °C for 30 min. The elution was collected by gravity-flow using a polypropylene column (Qiagen). Sample homogeneity and identity were evaluated using SDS-PAGE and LC-MS (NHLBI Biochemistry Core). Measured masses were: 14,461 Da for 1–140; 12,994 Da for 14–140; 10,869 Da for 36–140; 10,337 Da for 41–140. Protein concentrations were determined using a molar extinction coefficient estimated on basis of amino-acid content: $\varepsilon_{280\,nm}$ = 5,960 M^{-1} cm^{-1} for 1–140, 14–140, and 36–140 and $\varepsilon_{280\,nm}$ = 4,470 M^{-1} cm^{-1} for 41–140. All purified proteins were aliquoted and stored at −80 °C until use. All buffers were filtered (0.22 µm).

Fibril formation and ThT kinetics. Prior to aggregation, protein samples were exchanged into pH 7.4 buffer (20 mM NaPi, 140 mM NaCl) using a PD-10 desalting column (GE Healthcare) and filtered through YM-100 spin units (Millipore) to remove any preformed aggregates. For cryo-EM studies, fibrils were formed by aggregating 41–140 in microcentrifuge tubes (cat. 022431081, Eppendorf) containing 1-mL solution (180 µM) with continuous shaking at 600 rpm at 37 °C for 4–5 days in a Mini-Micro 980140 shaker (VWR). Fibrils were stored at RT until use. ThT-monitored aggregation reactions (40–70 µL, [protein] = 35–70 µM, [ThT] = 3.5–10 µM) were carried out in sealed black, polypropylene, 384-well flat-bottom microplates (781209, Greiner Bio-one)

supplemented with 2-mm glass beads. Seeds (1–140, 14–140, 36–140, or 41–140) were prepared by ultracentrifugation (100,000 rpm, TLA100 rotor, Beckman Coulter, for 45 min at 4 °C) from aggregation reactions (100 μM). Then, the pelleted seeds were quantified by absorbance measurements after GuHCl dissolution (3 M final). Seeds (0.7–1.4 μL, 100 μM) were added to a solution of 35 μM 1–140, 14–140, 36–140, or 41–140, to a final volume of 40 μL. Fluorescence (excited and monitored at 415 and 480 nm, respectively) was recorded at 37 °C with continuous linear shaking (1 mm) using a microplate reader (Tecan Infinite M200 Pro). A total of at least 2 independent experiments were performed with at least 4 replicates for each condition on each plate. For SDS-PAGE analysis of soluble and insoluble fractions, separation was achieved by ultracentrifugation (100,000 rpm, TLA100 rotor, Beckman Coulter) for 45 min at 4 °C. Densitometric analysis was calculated from SDS-PAGE gels using the image processing package, Fiji.

TEM. Samples (10 μL) were applied to TEM grids (400-mesh formvar and carbon coated copper, Electron Microscopy Sciences) for approx. 2 min and wicked away by filter paper. Deionized water (10 μL) was then applied and wicked away immediately. A solution of 1% uranyl acetate (10 μL) is placed on the grid for 2 min, wicked away, and air-dried. TEM was performed using a JEOL JEM 1200EX transmission electron microscope (accelerating voltage 80 keV) equipped with an AMT XR-60 digital camera (NHLBI EM Core Facility). Fibril helical twists were calculated using the image processing package, Fiji.

Degradation reactions of α-syn fibrils by Proteinase K. In glass vials, various α-syn fibrils (45 μM) were incubated with Proteinase K (Invitrogen) at different concentrations (2 μg, 0.2 μg, 20 ng, 2 ng) in reaction buffer (20 mM NaPi, 140 mM NaCl, pH 7.4) in a total volume of 50 μL. Samples were agitated at 600 rpm for 20 h at 37 °C in a Mini-Micro 980140 shaker (VWR). Reactions were terminated with 0.1% TFA and 3 M guanidinium hydrochloride.

LC-MS. Proteolyzed samples (5 μL) were separated using a HPLC (Agilent 1100 series HPLC, Agilent Technologies) on a reversed-phase C18 column (Zorbax, 2.1 x 50 mm, 3.5 μm, Agilent Technologies). Peptides masses of α-syn were obtained with an Agilent 6224 electrospray ionization time-of-flight LC-MS. For mobile phase, a gradient (0–50% acetonitrile and 0.05% TFA) at a flow rate of 0.2 mL/min was used. The HPLC systems and MSD were controlled and data analyzed using the Agilent MassHunter Workstation platform. Mass spectra were obtained using positive ion mode. Data for reactions with 2 ng PK are shown in **Table S2**.

Mammalian cell culture. Human SH-SY5Y neuroblastoma cells were maintained in Dulbecco's Modified Eagle Medium (DMEM) supplemented with 4 mM L-glutamine, 10% FBS, and 1% penicillin/streptomycin. Rat N27 dopaminergic cells were maintained in Roswell Park Memorial Institute Medium 1640 (RPMI 1640) supplemented with 2 mM L-glutamine, 10% FBS, and 1% penicillin/streptomycin. Cells were grown in a 37 °C incubator with 5% CO_2 and the media was replaced every 2–3 days. Cells were passaged with 0.25% Trypsin-EDTA (cat. no. 25200056, ThermoFisher) at 70–90% confluency.

MTT assay. SH-SY5Y and N27 cells (200 μL) cells at approximately 75,000 cells/mL were plated in each well of a 96-well plate (cat. no. CLS3599, Sigma) and allowed to attach overnight in a 37 °C incubator with 5% CO_2. The media was then replaced with media containing 1 μM of pre-formed fibrils that were resuspended in phosphate buffered saline (PBS). Negative and positive controls were media containing 1% PBS in the absence and presence of 1 mM H_2O_2, respectively. After 48 h, the media was replaced with media containing 0.5 mg/mL (3-(4,5-dimethylthiazol-2-yl)-2,5-diphenyl tetrazolium bromide) (MTT) and incubated for 2 h at RT. Cells were washed with

PBS, and the formazan product was completely dissolved in neat dimethyl sulfoxide before reading the absorbance at 590 nm on a Spark multimode plate reader (Tecan).

Cryo-EM specimen preparation and data collection. Fibril sample solutions (3 μL) were directly applied to freshly glow-discharged holey carbon grid (Quantifoil R1.2/1.3, copper, 300 mesh). The grids were blotted for 7 s and plunge frozen in liquid nitrogen-cooled liquid ethane using FEI Vitrobot Mark IV machine. Data were collected on a Titan Krios G3 electron microscope (Thermo-Fisher) operated at 300 kV and equipped with a Gatan Quantum LS imaging energy filter (GIF, energy filter slit width was set at 20 eV). Images were acquired on a K2 Summit direct detection camera at a magnification of 165,000× with a resulting pixel size of 0.83 Å using Leginon (4) software package automatically. Dose fractionations with 50 frames in the counting mode were recorded for 6.25-s exposure with a total dose of ~55 electrons per square angstrom ($e^{-}/Å^{2}$) for each micrograph. Detailed data collection parameters are listed in **Table S3**.

Cryo-EM data processing. MotionCorr2 (5) was used to perform a beam-induced motion correction. CTFFIND4 (6) was used to estimate defocus parameters. The following 2D classification, 3D classification, and 3D refinement were performed with RELION 3.0 (7), using methods previously described (8). Filament segments were manually picked and extracted using a box size of 256 pixels and an inter-box distance of 29.4 pixels (center-to-center). For 41–140, a total of 20,867 segments with 314,528 particles were extracted from 734 selected micrographs. (**Table S3**). The regularisation parameter T = 10 was used to perform 2D classification. 2D class averages with a clear separation of β-strands were selected for further 3D reconstructions.

Power spectra of 2D class averages show a strong layer line at 4.8 Å that corresponds to the helical rise (**Figure S10**). From the 2D class averages, 41–140 contained two asymmetrical protofilaments. The helical rise between two protofilaments is 4.8 Å. An additional 2D classification with segments extracted from a very large box size (1500 pixels) was performed to determine the approximate pitches of the α-syn fibrils. Considering the image features are repeated at a distance of half pitch, the length of half pitch was obtained by measuring the distance between two adjacent repeats. The initial helical twists were predicted from these pitches with the above deduced helical rise.

3D classification was performed using a cylinder generated *via* the Relion helix toolbox and low-pass filter to 50 Å as the initial model. 3D classification was started with a single class (K = 1) with the regularization parameter T = 4 and a central Z length of 10%, which defines the central part of the helical reconstruction. The resulting density map was used as reference for further 3D classifications with 4–6 classes. For the reconstruction of 41–140, series of 3D classification was performed until a cross-section along the *x*-*y* plane of the 3D reconstruction clearly showed zig-zagged features formed by the β-strands of the α-syn fibrils. For the reconstruction of 41–140, 44,161 particles were selected through the final 3D classification and subjected to the final 3D refinement with optimized helical symmetry. Because the available structures of α-syn fibrils solved by cryo-EM are conserved left-handed helices (9-12), the final helical twist here for 41–140 was optimized to −1.64° (**Table S3**). Post-processing was carried out with a soft-edge mask and an estimated sharpening B-factor of −100 $Å^{2}$ for 41–140. Bayesian polishing and CTF refinement were performed before the final post-processing step using RELION 3.0. The overall resolution was 3.2 Å for 41–140 (by the FSC 0.143 criterion, **Figure S11**).

Model building and refinement

The atomic model of 41–140 fibrils was *de novo* built in COOT (13). The high-quality EM maps allowed unambiguously assignment of residues E46–K96 for one protofilament and E61–D98 for the other protofilament. The resulting models (12 monomers with 6 adjacent layers) were refined against the EM maps using the real space refinement in PHENIX (14). The statistics are

summarized in **Table S3**. Buried surface area and total binding energy were estimated by using the jsPISA program (http://www.ccp4.ac.uk/pisa/).

References

1. Jakes R., Spillantini M.G., & Goedert M. Identification of 2 distinct synucleins from human brain. *FEBS Lett.* **345**, 27-32 (1994).
2. Watson M.D. & Lee J.C. N-terminal acetylation affects α-synuclein fibril polymorphism. *Biochemistry* **58**, 3630-3633 (2019).
3. Masuda M., *et al.* Cysteine misincorporation in bacterially expressed human α-synuclein. *FEBS Lett.* **580**, 1775-1779 (2006).
4. Suloway C., *et al.* Automated molecular microscopy: the new Leginon system. *J. Struct. Biol.* **151**, 41-60 (2005).
5. Zheng S.Q., *et al.* MotionCor2: anisotropic correction of beam-induced motion for improved cryo-electron microscopy. *Nat. Methods* **14**, 331-332 (2017).
6. Rohou A. & Grigorieff N. CTFFIND4: Fast and accurate defocus estimation from electron micrographs. *J. Struct. Biol.* **192**, 216-221 (2015).
7. Zivanov J., *et al.* New tools for automated high-resolution cryo-EM structure determination in RELION-3. *eLife* **7**, (2018).
8. He S. & Scheres S.H.W. Helical reconstruction in RELION. *J. Struct. Biol.* **198**, 163-176 (2017).
9. Guerrero-Ferreira R., *et al.* Cryo-EM structure of α-synuclein fibrils. *Elife* **7**, 18 (2018).
10. Li Y.W., *et al.* Amyloid fibril structure of α-synuclein determined by cryoelectron microscopy. *Cell Res.* **28**, 897-903 (2018).
11. Li B.S., *et al.* Cryo-EM of full-length α-synuclein reveals fibril polymorphs with a common structural kernel. *Nat. Commun.* **9**, 10 (2018).
12. Ni X.D., McGlinchey R.P., Jiang J.S., & Lee J.C. Structural insights into α-synuclein fibril polymorphism: Effects of Parkinson's disease-related C-terminal truncations. *J. Mol. Biol.* **431**, 3913-3919 (2019).
13. Emsley P. & Cowtan K. COOT: model-building tools for molecular graphics. *Acta Crystallogr. D Biol. Crystallogr.* **60**, 2126-2132 (2004).
14. Adams P.D., *et al.* PHENIX: a comprehensive Python-based system for macromolecular structure solution. *Acta Crystallogr. D Biol. Crystallogr.* **66**, 213-221 (2010).

Table S1. List of primers used to generate N-terminal truncations.

14–140 forward:
5’ AAAACATATGCATCACCATCATCACCACGAAAACTTATATTTCCAAGGAGTTGTGGCTGCTGC 3’

36–140 forward:
5’ AAAACATATGCATCACCATCATCACCACGAAAACTTATATTTCCAAGGTGTTCTCTATGTAGGCTCC 3’

41–140 forward:
5’ AAAACATATGCATCACCATCATCACCACGAAAACTTATATTTCCAAGGCTCCAAAACCAAGGAGGG 3’

Universal reverse:
5’ TGGCTCGAGTTAGGCTTCAGGTTCGTAGTC 3’

Table S2. MS analysis of PK digestion of 1–140, 14–140, 36–140 and 41–140 fibrils.

1–140 fibrils (45 μM) +Proteinase K (2 ng total)

Observed Mass (Da)	Theoretical Mass (Da)	Position in α-syn sequence
14460.75	14460.16	1–140
12705.20	12704.36	1–125
12597.39	12596.89	19–140
11413.06	11412.58	31–140
10841.65	10841.11	19–125
10770.52	10770.03	20–125
9657.19	9656.79	31–125

14–140 fibrils (45 μM) +Proteinase K (2 ng total)

Observed Mass (Da)	Theoretical Mass (Da)	Position in α-syn sequence
12994.85	12994.37	14–140
12597.46	12596.89	19–140
11413.14	11412.58	31–140
11239.05	11238.58	14–125
10770.54	10770.03	20–125
9657.17	9656.79	31–125
9113.60	9113.17	36–125

36–140 fibrils (45 μM) +Proteinase K (2 ng total)

Observed Mass (Da)	Theoretical Mass (Da)	Position in α-syn sequence
10869.45	10868.96	36–140
9113.49	9113.17	36–125
7737.03	7736.76	36–113

41–140 fibrils (45 μM) +Proteinase K (2 ng total)

Observed Mass (Da)	Theoretical Mass (Da)	Position in α-syn sequence
10337.69	10337.30	41–140
9314.43	9314.15	51–140
8582.05	8581.52	41–125
7558.82	7558.36	51–125
7205.61	7207.11	41–113

Table S3. Cryo-EM data collection, structure determination, and model statistics.

	**41–140**
**Data collection**	
Magnification	165,000*x*
Defocus range (μm)	−0.8 to −1.8
Voltage (kV)	300
Camera	Gatan K2 Summit
Microscope	Titan Krios
Exposure time (s/frame)	0.125
Number of frames	50
Total dose ($e^{-}/Å^2$)	55
Pixel size (Å)	0.83
**Reconstruction**	
Micrographs selected	734
Manually picked segments	20,867
Box size (pixel)	256
Inter-box distance (Å)	29.4
Particles extracted	314,528
Particles after Class2D	57,708
Particles after Class3D	44,161
Final resolution (Å)	3.2
Map sharpening B-factor ($e^{-}/Å^2$)	−100
Helical rise (Å)	4.8
Helical twist (°)	-1.64
**Atomic model**	
Number of protein residues	534
**Ramachandran plot values**	
Most favored (%)	86.67
Allowed (%)	13.32
Outliers (%)	0
Rotamer outliers	17.82
r.m.s.d Bond lengths (Å)	0.004
r.m.s.d Bond angles (°)	0.680
Clashscore	29.36
Map CC (main chain)	0.73
MAP CC (side chain)	0.72

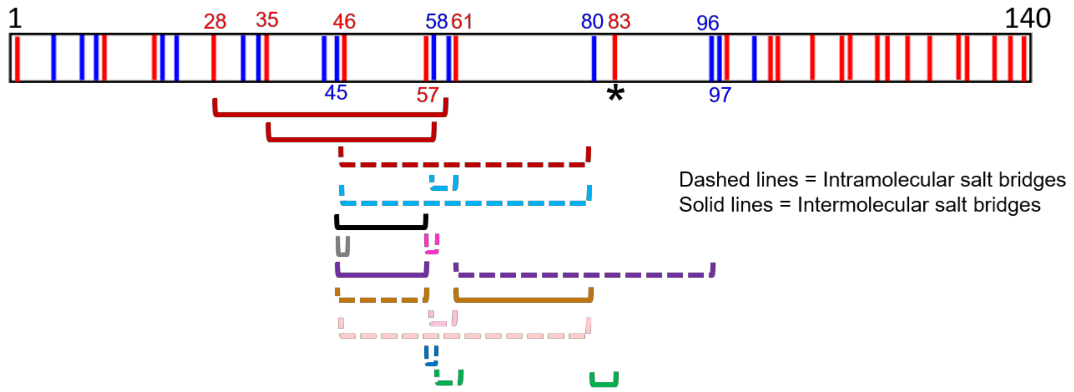

<u>Intermolecular salt bridges</u>

Ac1–140 (6XYO, 6XYP, 6XYQ) (E28–K60, E35–K58)
1–140 (6rt0) (K45–E57)
1–140 (6rtb) (K45–E46)
E46K (6UFR) (K45–E57)
E46K (6L4S) (E61–K80)
41–140 (K80–E83)

<u>Intramolecular salt bridges</u>

Ac1–140 (6XYO, 6XYP, 6XYQ) (E46–K80)
Ac1–140 and 1–140 (6OSJ, 6CU7, 6A6B) (E46–K80)
Ac1–140 (6OSJ, 6A6B) (K58–E61)
1–140 (6CU8) (E57–K58)
E46K (6UFR) (E61–K96)
E46K (6L4S) (K45–E57)
H50Q (6PES) (E46–K80 and K58–E61)
AcA53T (6LRQ) (E46–K80 and K58–E61)
PhosY39 (6L1T) (E57–K58)
41–140 (K58–E61)

Figure S1. Schematic representation of α-syn primary sequence (residues 1 to 140), showing basic (blue) and acidic (red) residues. Location of salt bridges identified in published cryo-EM structures of α-syn fibril variants (PDB codes) are shown as either inter- (solid lines) or intra-molecular salt bridges (dashed lines). Asterisk denotes salt bridge residues involved in 41–140 structure, that have not been previously used in other published structures.

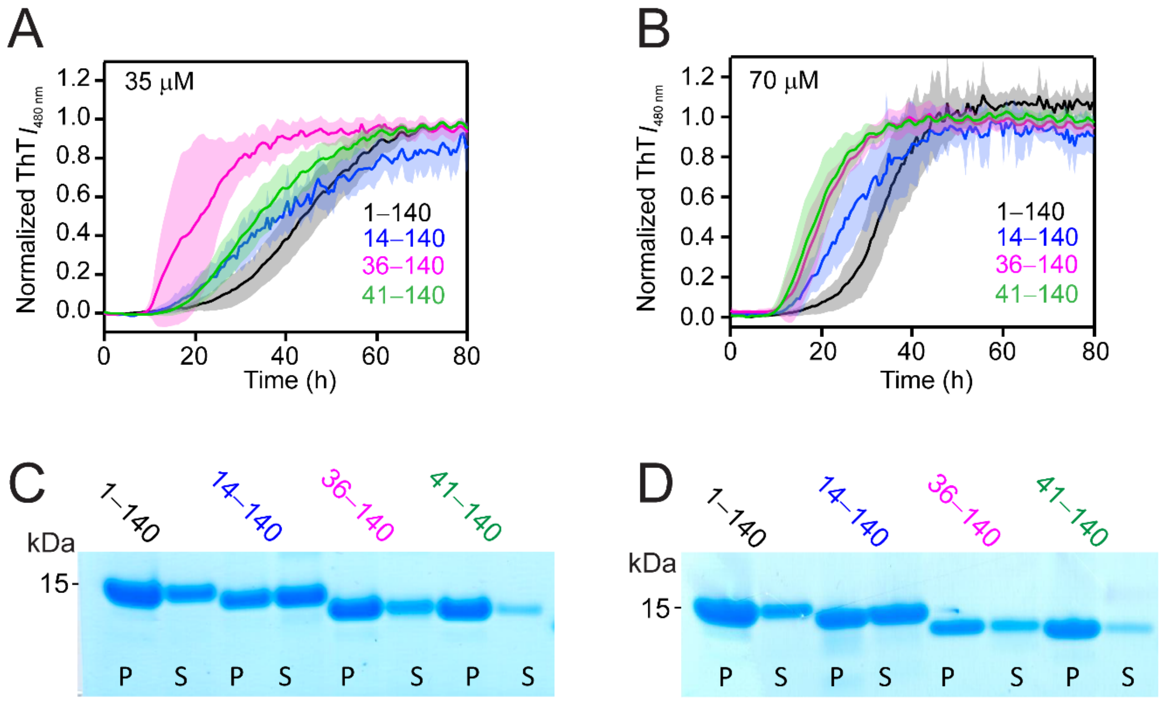

Figure S2. Comparison of normalized aggregation kinetics monitored by ThT fluorescence at 37 °C. [α-Syn] = 35 μM (**A**) and 70 μM (**B**) with [ThT] = 10 μM in 20 mM NaPi, 140 mM NaCl, pH 7.4. Solid line and shaded region represent the mean and standard deviation, respectively ($n \geq 4$). SDS-PAGE analysis of pelleted (P) and soluble (S) protein by ultracentrifugation for aggregation reactions conducted at (**C**) 35 μM and (**D**) 70 μM.

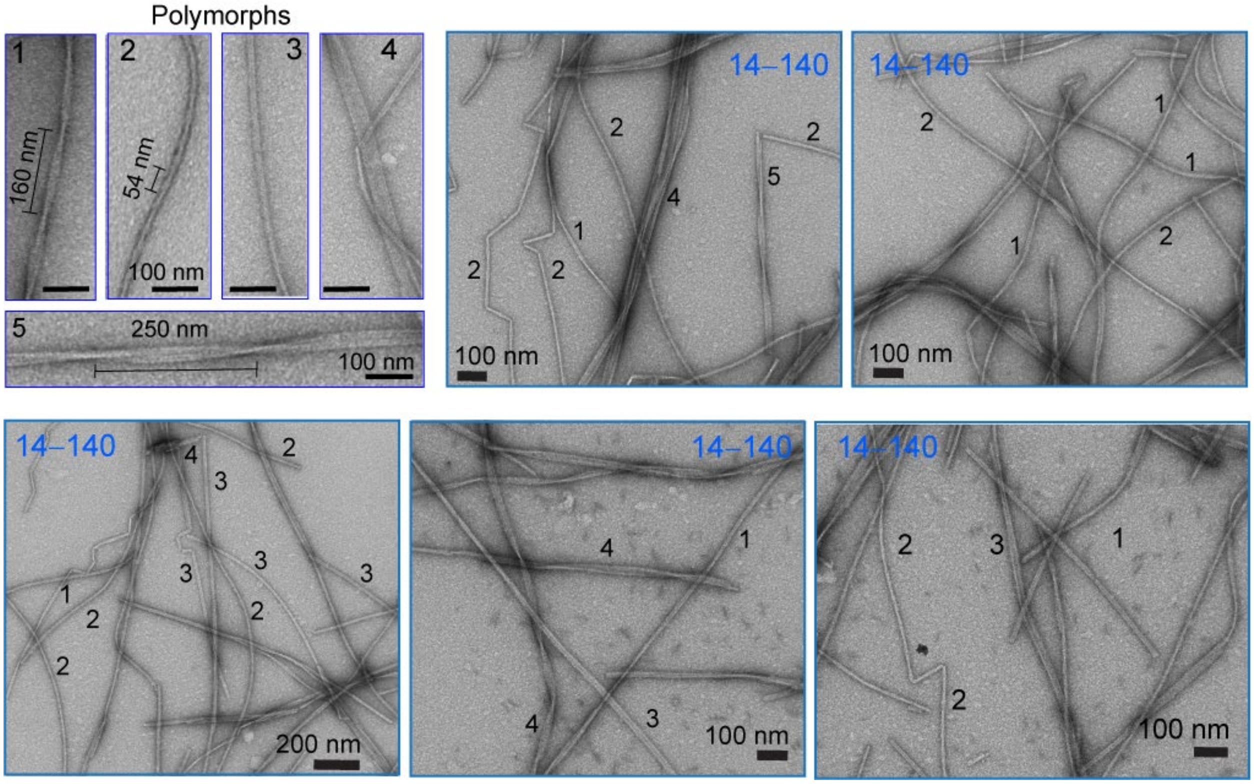

Figure S3. Negative-stain TEM images of 14–140 fibrils formed at pH 7.4. Highlighted are five different polymorphs that are either paired protofilaments that twist (polymorph 1 and 2) with varying half pitches (~54 and ~160 nm), rod (polymorph 3) or laterally associated protofilaments (polymorph 4) that can also bundle and twist (polymorph 5) with a helical pitch of ~250 nm. Scale bar is as shown.

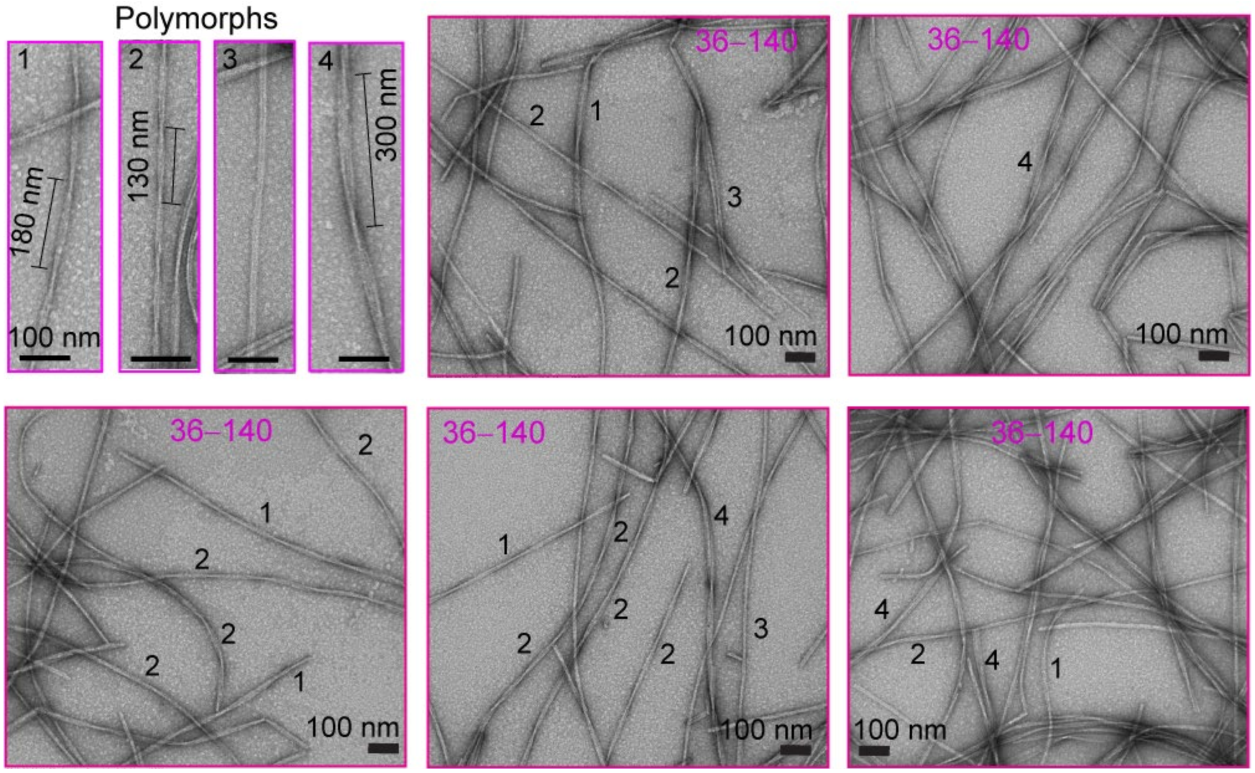

Figure S4. Negative-stain TEM images of 36–140 fibrils formed at pH 7.4. Highlighted are four different polymorphs that are either paired protofilaments that twist (polymorph 1 and 2) with varying half pitches (~180 and ~130 nm), rod (polymorph 3) and bundled protofilaments that twist (polymorph 4) with a half pitch of ~300 nm. Scale bars are 100 nm.

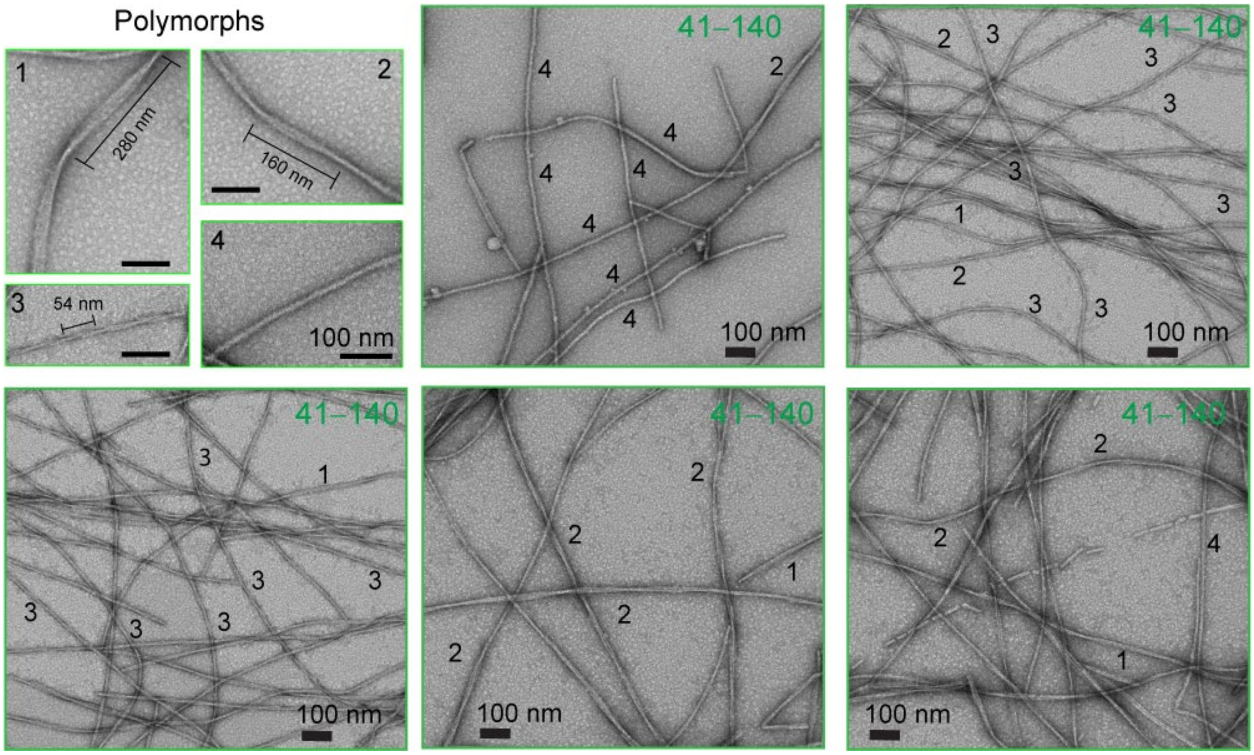

Figure S5. Negative-stain TEM images of 41–140 fibrils formed at pH 7.4. Highlighted are four different polymorphs that are bundled protofilaments that twist (polymorph 1 and 2) with half pitches of ~280 and ~160 nm. Paired protofilaments that twist (polymorph 3 and 4) with an infrequently observed half pitch of ~54 nm. Scale bars are 100 nm.

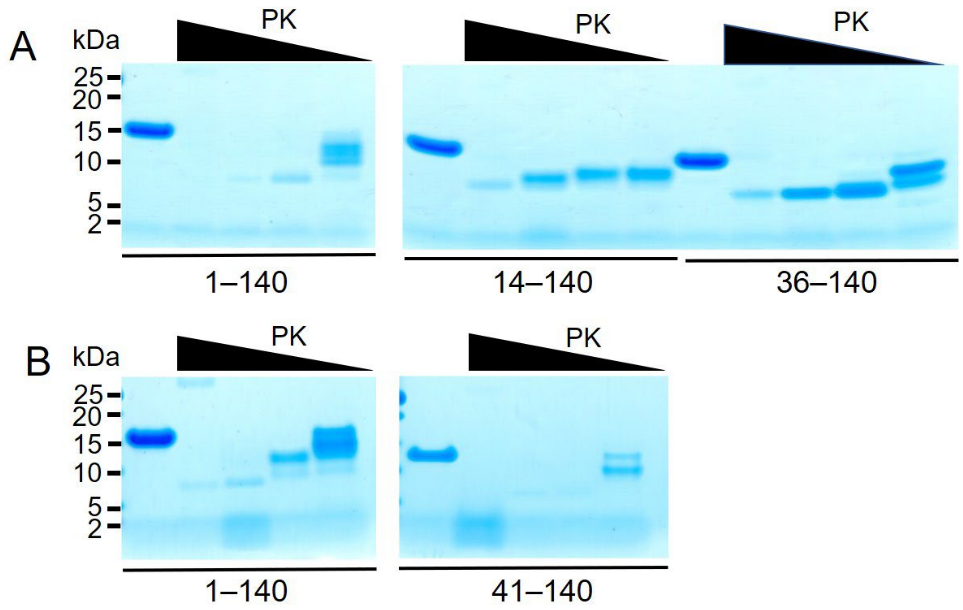

Figure S6. SDS-PAGE (4–12%) analysis of proteinase K (PK) degradation of 1–140, 14–140, 36–140, 41–140, and 66–140. Fibrils (45 µM) were incubated with decreasing PK (2 µg, 0.2 µg, 20 ng, and 2 ng) for 20 h at 37 °C. Degradation profiles of 1–140 are compared to 14–140 and 36–140 (**A**), and 41–140 (**B**). Samples (20 µL + 10 µL LDS loading buffer) were boiled for 15 min, and gels are visualized by SimplyBlue™ SafeStain (Thermo Fisher Sci.).

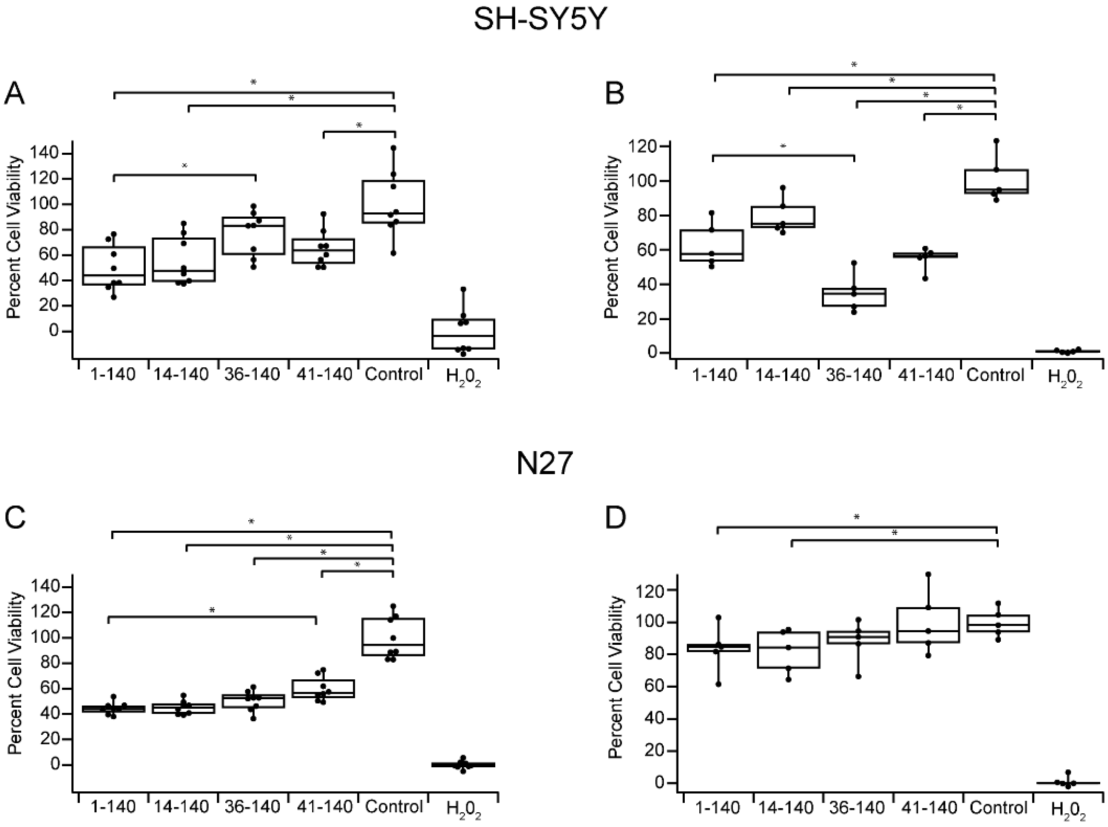

Figure S7. Biochemical analysis of 1–140 *vs.* ΔN-α-syn fibrils. SH-SY5Y (**A** and **B**) and N27 (**C** and **D**) cells were treated with 1 μM fibril for 48 h before cell viability was determined *via* a MTT assay. MTT response was normalized to the controls of media in the presence and absence of 1 mM H_2O_2, representing live and dead cells signals. Fibril-treated samples showed significantly reduced cell viability compared to the negative control (n = 8 in panels **A** and **C**; n = 5 in panels **B** and **D**). Asterisks denote $P < 0.05$ based on a 2-tailed students T-test comparing fibrils to the control and between fibril samples.

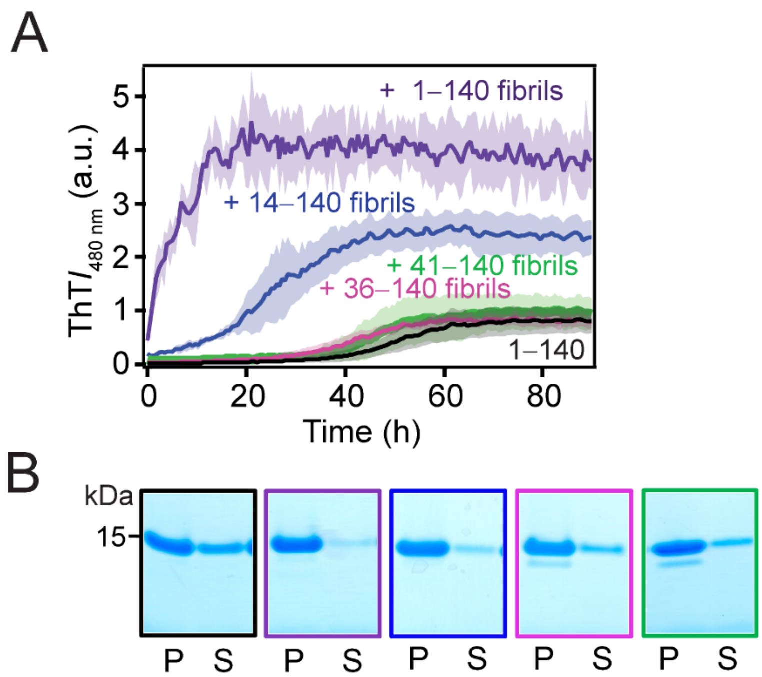

Figure S8. Cross-seeding kinetics of 1–140 with ΔN-α-syn fibrils. (**A**) Aggregation kinetics of 1–140 in the absence (black) and presence of pre-formed 1–140 (purple), 14–140 (blue), 36–140 (magenta), or 41–140 (green) fibrils (10% mol/mol) monitored by ThT fluorescence ([1–140] = 35 μM and [ThT] = 3.5 μM in 20 mM NaPi, 140 mM NaCl, pH 7.4 at 37 °C). Solid line and shaded region represent the mean and standard deviation, respectively ($n \geq 4$). (**B**) SDS-PAGE analysis of pelleted (P) and soluble (S) protein taken post-aggregation by ultracentrifugation. Colors correspond to those in panel A.

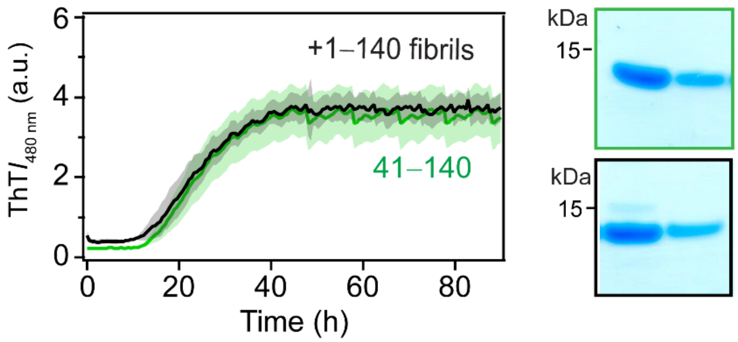

Figure S9. Cross-seeding kinetics of 41–140 with 1–140 fibrils. Aggregation kinetics of 41–140 in the absence and presence of 1–140 fibrils (10% mol/mol) monitored by ThT fluorescence (left, [41–140] = 35 μM and [ThT] = 3.5 μM in 20 mM NaPi, 140 mM NaCl, pH 7.4 at 37 °C). Solid line and shaded region represent the mean and standard deviation, respectively ($n \geq 4$). SDS-PAGE analysis of pelleted (P) and soluble (S) proteins by ultracentrifugation post-aggregation (right).

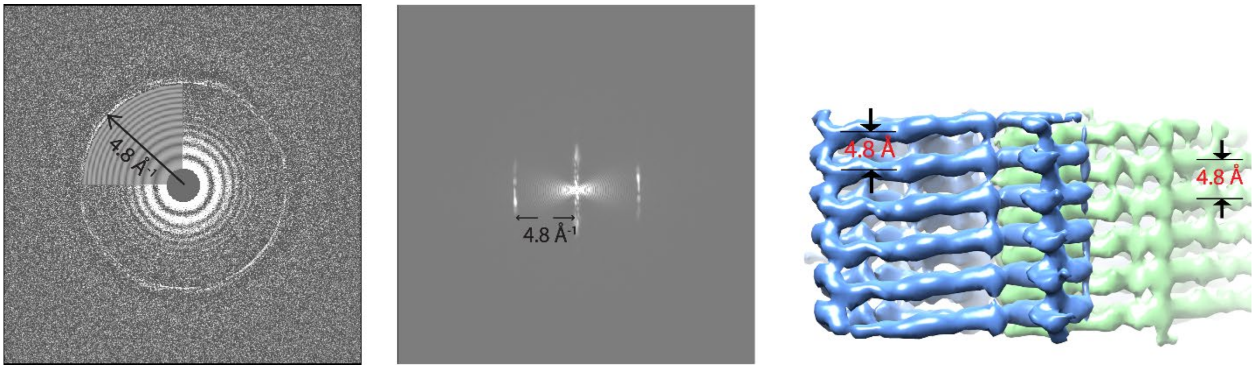

Figure S10. Helical parameter estimation. (*Left*) Representative power spectrum from a selected micrograph showing the combined layer lines (show with an arrow) from randomly oriented fibrils that correspond to the helical rise. (*Middle*) The layer lines calculated from a 2D class average are consistent with the rise calculated from power spectrum. (*Right*) A side view of the 41–140 density map with the helical rise labeled between two stacked β-sheets.

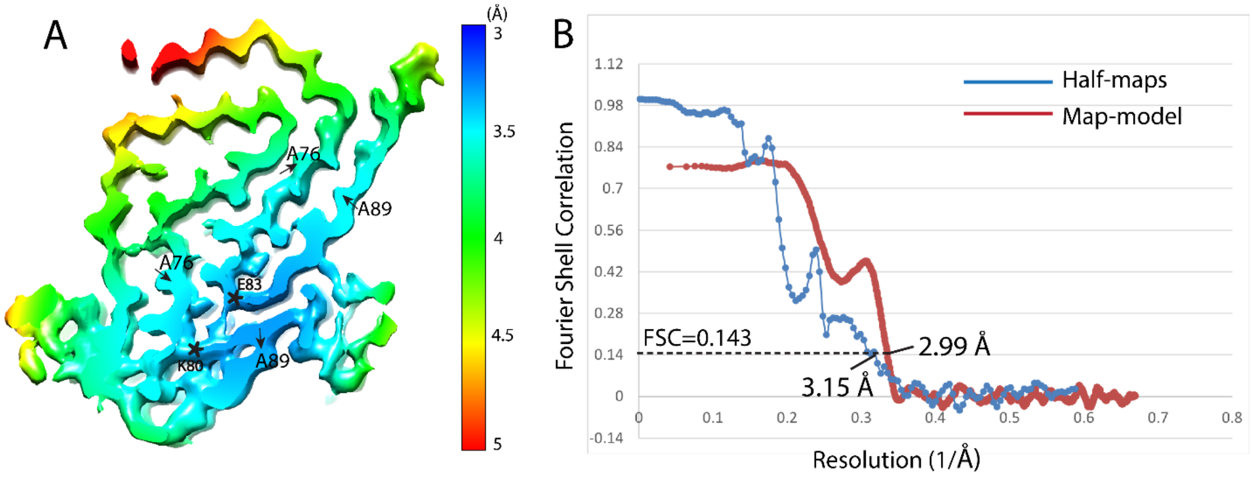

Figure S11. Local resolution estimation and gold-standard Fourier Shell Correlation (FSC) curves for resolution estimation for 41–140. (**A**) EM density map colored based on local resolution, showing the highest resolution around the interfacial region between the two protofilaments, especially between residues A76–A89, denoted by arrows. The intermolecular salt bridge K80–E83 is located within this region and labelled with asterisks. (**B**) An FSC cutoff value at 0.143 was used to estimate the final resolution and the map-model correlations for 41–140. The overall resolution is 3.2 Å.

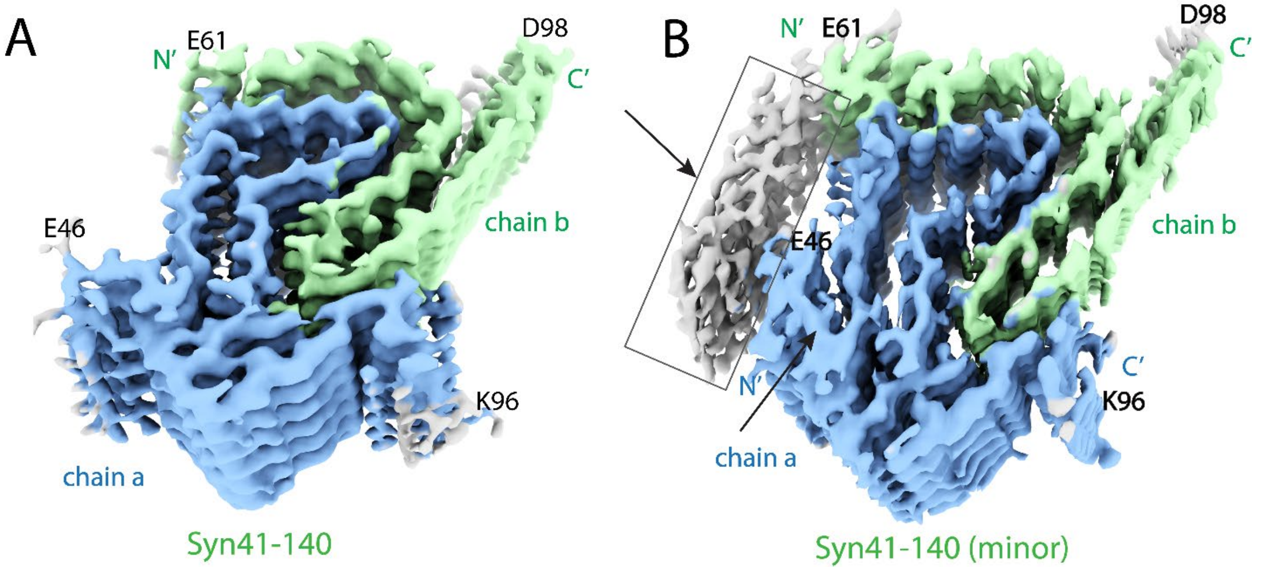

Figure S12. Two conformations of 41–140 structure. (**A**) Cryo-EM density map of the major conformation of 41–140. The fibril contains two asymmetrical protofilaments, chain a (blue) and chain b (green). Chain a consists of ordered residues E46–K96, while chain b contains E61–D98. (**B**) A minor conformation of 41–140 structure reveals additional density at the N-terminal region of chain b, as indicated by the rectangle. This low resolution density prevented residue assignments to be made, but likely represents residues from G41 to K60.

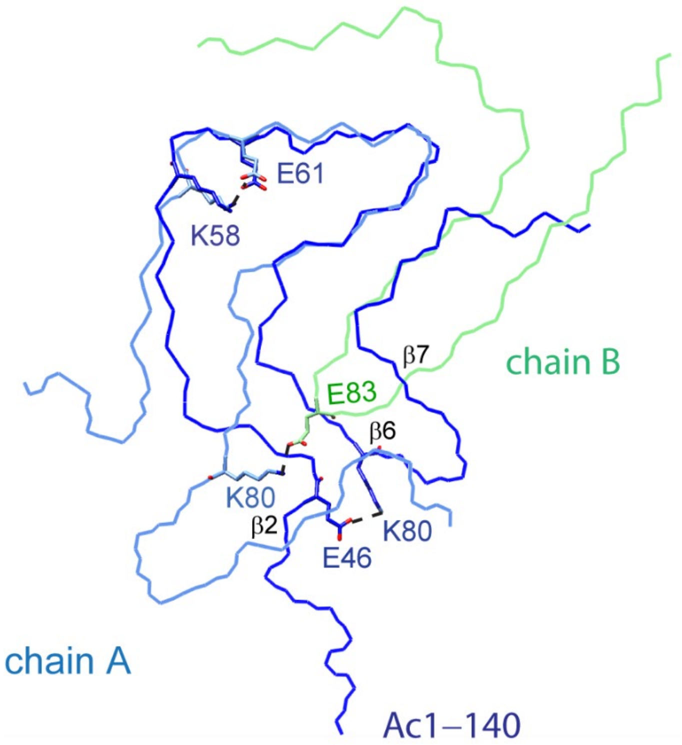

Figure S13. Overlay of Ac1–140 (PDB 6OSJ, blue) with chain A (light blue) and chain B (green) of 41–140 with β-strand assignments based on the Ac1–140 structure. Salt bridges E46–K80 and K58–E61 for Ac1–140, and K80–E83 and K58–E61 for 41–140 are shown.

.
